# A comparison of FreeSurfer-generated data with and without manual intervention

**DOI:** 10.3389/fnins.2015.00379

**Published:** 2015-10-21

**Authors:** Christopher S. McCarthy, Avinash Ramprashad, Carlie Thompson, Jo-Anna Botti, Ioana L. Coman, Wendy R. Kates

**Affiliations:** Department of Psychiatry and Behavioral Sciences, Center for Psychiatric Neuroimaging, State University of New York at Upstate Medical UniversitySyracuse, NY, USA

**Keywords:** FreeSurfer, intensity normalization, control points, white matter edits, interactive, semi-automatic

## Abstract

This paper examined whether FreeSurfer—generated data differed between a fully—automated, unedited pipeline and an edited pipeline that included the application of control points to correct errors in white matter segmentation. In a sample of 30 individuals, we compared the summary statistics of surface area, white matter volumes, and cortical thickness derived from edited and unedited datasets for the 34 regions of interest (ROIs) that FreeSurfer (FS) generates. To determine whether applying control points would alter the detection of significant differences between patient and typical groups, effect sizes between edited and unedited conditions in individuals with the genetic disorder, 22q11.2 deletion syndrome (22q11DS) were compared to neurotypical controls. Analyses were conducted with data that were generated from both a 1.5 tesla and a 3 tesla scanner. For 1.5 tesla data, mean area, volume, and thickness measures did not differ significantly between edited and unedited regions, with the exception of rostral anterior cingulate thickness, lateral orbitofrontal white matter, superior parietal white matter, and precentral gyral thickness. Results were similar for surface area and white matter volumes generated from the 3 tesla scanner. For cortical thickness measures however, seven edited ROI measures, primarily in frontal and temporal regions, differed significantly from their unedited counterparts, and three additional ROI measures approached significance. Mean effect sizes for edited ROIs did not differ from most unedited ROIs for either 1.5 or 3 tesla data. Taken together, these results suggest that although the application of control points may increase the validity of intensity normalization and, ultimately, segmentation, it may not affect the final, extracted metrics that FS generates. Potential exceptions to and limitations of these conclusions are discussed.

## Introduction

FreeSurfer^1^ (FS) is a freely available fully automated brain image morphometric software package that allows for the measurement of neuroanatomic volume, cortical thickness, surface area, and cortical gyrification of regions of interest (ROIs) throughout the brain. FS was designed around an automated workflow that encompasses several standard image processing steps necessary to achieve a final brain parcellation within the subject's space; however, manual image editing is allowed after each stage to ensure quality control. The first stage performs skull stripping and motion artifact correction, the second performs gray-white matter segmentation (Fischl et al., 2002), and the third segments 34 ROIs based on anatomic landmarks (Desikan et al., 2006). Another critical function that FS provides is the ability to construct surface-based representations of the cortex, from which cortical thickness, neuroanatomic volume, and surface area can be derived. Manual measurement of the volumes of specific ROIs is an arduous, labor-intensive task, and is subject to inter-rater variability. FS offers consistency in its fully automated processing, which is ideal for either single- or multi-site studies with large sample sizes. In general, validation studies have demonstrated that FS can produce measurements that are comparable to those derived from manual tracing of brain regions (Fischl et al., 2002; Tae et al., 2008; Bhojraj et al., 2011). FS has also been shown to be a highly reliable method for automated cortical thickness measurements across scanner strength and pulse sequence in all regions of the brain, with minor variability being attributed to cytoarchitectural differences of certain ROIs and difficulties with surface reconstructions in temporal lobe regions (Han et al., 2006; Fjell et al., 2009).

However, strictly implementing the automated procedures in FS can result in variability in the accuracy of segmentation for some ROIs. For example, Cherbuin et al. (2009) showed that absolute hippocampal volumes measured with FS were significantly larger than those of manual tracings, with reported 23 and 29% overestimation of left and right hippocampal volumes, respectively. Closer inspection revealed that this was due to inclusions of surrounding high intensity voxel structures as well as misidentification of pockets of cerebrospinal fluid as hippocampal tissue (Cherbuin et al., 2009). Other studies suggest that the temporal lobe and nearby regions are troublesome areas of the brain for FS to measure accurately (Desikan et al., 2006; Oguz et al., 2008). The presence of either excess dura matter, closely adjacent temporal bone or cerebellum can potentially lead to inclusions which may affect volume and ROI segmentation (Desikan et al., 2010). Moreover, some neuropathological conditions, which lead to enlarged ventricles like normal pressure hydrocephalus or Alzheimer's disease may affect white matter segmentation steps and thus may lead to greater necessity of editing the FS images of patients with similar conditions (Moore et al., 2012). Magnetic Resonance (MR) imaging acquisition artifacts can also lead to over-inclusion of white matter.

Given the propensity of FS to include areas of the brain extraneous to the ROI, investigators have the option of interrupting the automated process and its output. This can be done via skull stripping the brain, via the addition of control points to correct intensity normalization, via direct manual edits of white matter boundaries, or via a combination of these manual editing methods. These manual edits alter the white matter surface so that it more fully includes white matter structures and does not mistakenly segment gray matter or non-brain tissue as white matter. Manually editing the skull strip can ensure that it is more precise than the automatically completed procedure implemented by FS, and not affected by altered local anatomy in pathological states (Fennema-Notestine et al., 2006). This may improve the segmentation of white matter and lead to less control point placement in the next stage of quality control human intervention.

We reviewed 82 previous studies published primarily between 2006 and 2013 (see Table 1) that utilized FS, discovering a great deal of variability in the extent to which investigators utilized skull stripping, control point or white matter editing options (see Table 1 for review criteria). Two of the studies obtained their samples from previously established databases. Of those 82 studies, 36 utilized 3 tesla (T) or higher MRI scanners, with 8 of those electing the fully automated procedure (31%). The remaining 18 chose to manually edit their 3T data using different combinations of skull stripping, control points, and white matter editing options (69%). The remaining studies utilized 1.5T MRI scanners with 26 choosing the fully automated procedure (46%). Thirty-one 1.5T studies implemented some combination of manual intervention (54%). Scanner strength did not robustly affect whether or not a study decided to edit their data. Fujimoto et al. (2014) compared 3T and 7T data, and reported only editing 7T data for residual hyperintensities in the temporal lobe while leaving the 3T unedited. Pfefferbaum et al. (2012) compared 3T data to 1.5T data, and chose to edit the 3T images more extensively. The heterogeneity in the papers we reviewed underlines the lack of a standard protocol for deciding whether to interrupt the FS segmentation process and manually edit.

**Table 1:** **Methodological variations in articles utilizing FreeSurfer, published between 2006 and 2013^a^**.

**Study (Year)**	**Patient *N***	**Pathology**	**Control *N***	**Total *N***	**Scanner type/Strength**	**Manual edits**
Alner et al., 2012	26	Insertional mutation	10	36	1.5T GE Signa	S, C
Anticevic et al., 2012	20	HC	0	20	3T Siemens Allegra	
Barnes et al., 2010	78	HC	0	78	1.5T GE Signa	S, C
Batty et al., 2010	25	ADHD (age 9–15)	24	49	1.5T Phillips Achieva	S, C, W
Benedict et al., 2009	50	MS	20	70	1.5T GE Signa	S
Bhojraj et al., 2011	56	SCZ	36	92	1.5T GE	S, C, W
Bomboi et al., 2011	24	MS	24	48	1.5T, 3T GE Signa	C, W
Bray et al., 2011	59	Fragile X	83	142	1.5T GE Signa	C
Cerasa et al., 2011	109	MAO-A alleles	0	109	3T Siemens Allegra	S, C, W
Cherbuin et al., 2009	403	HC	0	403	1.5T Phillips	
Chiang et al., 2011	149	HC (elderly)	0	149	1.5T	
Clarkson et al., 2011	106	AD and FTD	44	150	1.5T GE Signa	
Dalaker et al., 2011	43	PD	41	84	1.5T Philips Intera	S, C
Desikan et al., 2010	162	MCI	0	162	1.5T GE, Siemens, or Phillips	
Dickerson et al., 2007	15	HC	0	15	1.5T Siemens Avanto	
Du et al., 2007	41	AD and FTD	23	64	1.5T Siemens Vision	
Durand-Dubief et al., 2012	9	MS	0	9	1.5T Siemens Sonata + Intera	
Dykstra et al., 2012	5	TLE	0	5	T1 Weighted MRI	S, C
Eggert et al., 2012	38	Simulated	0	38	3T Siemens TRIO	
Ehrlich et al., 2012	131	SCZ	138	269	1.5T, 3T Siemens Sonata + TRIO	C
Eyler et al., 2011	202	HC (twins)	0	404	1.5T Siemens	C
Feczko et al., 2009	29	AD	76	105	1.5T Siemens	S, C, W
Fennema-Notestine et al., 2007	133	HC (elderly)	0	133	1.5T GE Signa + Siemens	C
Fennema-Notestine et al., 2009	259	AD and MCI	139	398	ADNI WEBSITE	C
Fennema-Notestine et al., 2006	16	Various diagnoses	0	16	1.5T GE	
Fjell et al., 2009	883	HC (18-93)	0	883	1.5T	S, C
Francis et al., 2011	70	Children of SCZ patients	73	143	1.5T GE	S, C, W
Fujimoto et al., 2014	6	HC	0	6	7T Siemens	C
Furst and Lal, 2011	13	AD	11	24	1.5T Siemens Vision	C
Goghari et al., 2007a	19	Relatives of SCZ patients	22	41	3T GE Signa	S, C
Goghari et al., 2007b	19	Relatives of SCZ patients	22	41	3T GE Signa	S, C, W
Goldman et al., 2009	307	SCZ and unaffected siblings	196	503	1.5T GE	
Gronenschild et al., 2012	10	Various diagnoses	20	30	3T Siemens Allegra	
Gutierrez-Galve et al., 2009	38	APO-E allele	23	61	1.5T GE Signa	S, C, W
Han et al., 2006	15	HC	0	15	1.5T Siemens Sonata + 3T Siemens	
Hinds et al., 2009	10	HC	0	10	3T TIM TRIO	C
Iglesias et al., 2011	92	HC	0	92	1.5T Siemens	
Keller et al., 2012	10	JME	62	72	3T Philips Intera	
Khan et al., 2008	50	Various diagnoses	0	50	1.5T GE + Siemens	
Klein and Tourville, 2012	101	HC	0	101	3T, 7T	C
Kremen et al., 2010	474	HC (twins)	0	474	1.5T Siemens	C
Lee et al., 2006	30	Cases from ICBM	0	30	Database	C
Lehmann et al., 2010	51	AD and FTD	25	76	1.5T GE	S, C
Levinski et al., 2009					1.5	S, C, W
Mahone et al., 2011	42	ADHD	44	86	1.5T Phillips	
Makris et al., 2007	24	ADHD (adults)	18	42	1.5T Siemens Sonata	
Moore et al., 2012	15	NPH and AD	15	30	3T GE Signa	C
Morey et al., 2009	20	HC	0	20	3T GE Excite	
Mueller et al., 2010	38	AD and MCI	53	91	4T BrukerMedSpec	S
Murakami et al., 2011	21	SCZ	21	42	3T GE Signa	S, C
Nesvåg et al., 2009	53	SCZ/SCA	0	53	1.5T GE	C
Noble et al., 2012	60	HC	0	60	1.5T Siemens Sonata	S, C
O'Donnell et al., 2005	35	HC (8–20)	0	35	1.5T GE Horizon	S, C
Oertel-Knöchel et al., 2013	54	SCZ and healthy relatives	37	91	3T Siemens Allegra	S, C, W
Oguz et al., 2008	9	HC	0	9	1.5T	
Ostby et al., 2009	171	HC	0	171	1.5T Siemens Avanto	S, C
Panizzon et al., 2009	474	Publicly available images	0	474	1.5T Siemens	S, C
Park et al., 2004	1	Tumor	1	2	1.5T	S, C
Park et al., 2009	33	Blind from birth or after	35	68	3T Philips	S, C
Pellicano et al., 2010	24	MS	24	48	3T GE Signa	C
Pengas et al., 2009	11	SD	0	11	1.5T GE Signa	
Pfefferbaum et al., 2012	114	HC	0	114	1.5T, 3T GE Signa	C
Poulin et al., 2011	367	AD from ADNI base	0	367	1.5T GE, Siemens, or Phillips	
Putcha et al., 2011	16	MCI	18	34	3T Siemens Trio	S
Raj et al., 2010	27	Drug resistant TLE	30	57	4T BrukerMedSpec	C
Ramasamy et al., 2009	88	MS	38	126	1.5T GE Signa	C, W
Rimol et al., 2010	312	SCZ and BD	207	519	1.5T Siemens Sonata	
Rohrer et al., 2009	76	SD and aphasia	29	105	1.5T GE Signa	S, C, W
Romero-Garcia et al., 2012	30	HC elderly	0	30	1.5T Phillips Intera	C
Safford et al., 2010	13	HC	0	13	3T Siemens Allegra	
Schultz et al., 2010b	59	SCZ	59	118	1.5T Siemens Vision	
Schultz et al., 2010a	54	SCZ	54	108	1.5T Siemens Vision	
Shattuck et al., 2009	40	HC	0	40	1.5T GE	
Strangman et al., 2010	50	TBI	0	50	1.5T Siemens Avanto	
Tae et al., 2008	21	MDD	20	41	1.5T Philips	
Tomasevic et al., 2013	20	RRMS	0	20	1.5T Philips	S, C
Tosun et al., 2010	171	AD and MCI	77	248	1.5T	
Travis et al., 2014	17	HC (infants)	0	17	1.5T GE	C
Weier et al., 2012	15	MS	15	45	1.5T Siemens Avanto	
Winkler et al., 2010	486	HC	0	486	3T Siemens Trio	
Wonderlick et al., 2009	11	HC	0	11	3T Siemens Trio	S
Woodward et al., 2009	50	PTSD	47	97	1.5T GE Signa	

a The literature review was conducted during the summer of 2013 via PubMed search of the terms “freesurfer” and “freesurfer editing,” which yielded hundreds articles largely between the years 2006 and 2013. Studies were excluded (1) if they were based on dissertations, posters, or abstracts; or (2) if they compared FreeSurfer performance between computer systems, as most did not report utilizing any manual editing protocol. The full length studies remaining had their methods section analyzed for the precise methods of editing used. Studies which definitively stated that no editing was done were included, as it showed awareness of the possibility to edit. If they failed to mention manual editing in their methods section, they were excluded on the basis of the wide variability in possible minor manual edits that could have been made and gone unreported. Additionally, if the study did mention minor manual intervention, but failed to specify precisely what was done at each stage, the study was excluded. We also looked for studies that applied homogenous editing protocols to all study scans: those in which only a subset of scans were edited were excluded due to variability in selection of the images to be edited.

AD, Alzheimer's; PTSD, post-traumatic stress disorder; ADHD, attention-deficit hyperactivity disorder; RRMS, relapsing-remitting MS; BD, bipolar disorder; SCA, schizoaffective disorder; FTD, frontotemporal dementia; SCZ, schizophrenia; HC, healthy controls; SD, semantic dementia; JME, juvenile myoclonic epilepsy; TBI, traumatic brain injury; MDD, major depressive disorder; TLE, temporal lobe epilepsy; MS, multiple sclerosis; S, data was manually skull stripped; MCI, mild cognitive impairment; C, control points were manually applied; NPH, normal pressure hydrocephalus; W, white matter edits were utilized; PD, Parkinson's disease.

Given that there is no standard protocol for the decision to interrupt the fully automated FS pipeline to manually edit the images, this paper seeks to establish the extent to which editing affects the final measurements that FS provides. Conceivably, time consuming manual interventions may only marginally affect the edited data sets, leading one to believe that the editing of this data may only be necessary for specific ROIs. To that end, our study is constructed around the following question: To what extent do the FreeSurfer-generated data for each region of interest differ significantly between the edited and unedited (i.e., fully automated) methods of measurement? Accordingly, we compare the means and variances of surface area, white matter volumes, and cortical thickness derived from edited and unedited datasets for each of the 34 ROIs. Note that surface area was chosen instead of gray matter volume, since surface area has been shown to be genetically and phenotypically independent of cortical thickness (Panizzon et al., 2009; Winkler et al., 2010) and, therefore, more informative than gray matter volume. Moreover, we compare effect sizes between edited and unedited conditions in a small sample of individuals with 22q11.2 deletion syndrome (22q11DS) and neurotypical controls, in order to determine whether or not editing FS output would alter the sample size necessary to detect significant differences in surface area, white matter, or cortical thickness. We hypothesize that the values generated by the edited method will differ from those of the unedited method, and that the edited method will produce larger effect sizes.

## Materials and methods

### Participants

Data used in this study were selected from an ongoing longitudinal study focusing on biomarkers for psychosis in 22q11.2 deletion syndrome (Kates et al., 2011a). The procedures of the longitudinal study were approved by the Institutional Review Board at SUNY Upstate Medical University. Participants were recruited through the SUNY Upstate International Center for the Evaluation, Treatment and Study of Velo-Cardio-Facial Syndrome and from the community, and all participants provided informed consent. Imaging data and neuropsychiatric testing data were acquired at four visits, about 3 years apart. For the first three time points, images were acquired on a 1.5T scanner; for the fourth time point, images were acquired on a 3T scanner.

The subsample with imaging data from the 1.5T MR scanner was drawn from a larger sample of 116 participants who returned for the third time point of the longitudinal study. The subsample consisted of the first 30 participants (stratified by study group) whose Time 3 imaging data were processed, roughly corresponding to the order in which the participants returned for Time 3. They consisted of 20 with 22Q11.2 deletion syndrome (22q11DS) (8 male; mean age 17.54, SD 1.9) and 10 community controls (4 male; mean age 17.18, SD 1.21).

The subsample of participants whose imaging data was from the 3T MR scanner consisted of 21 subjects who returned for the fourth time point and had been included in the subsample with 1.5T MR dataset. Nine additional subjects were matched by age, gender, and diagnosis to the remaining participants from the 1.5T MR subsample. The mean age of the 22q11DS group was 20.74, SD 2.1, and the mean age of the control group was 20.42, SD 1.06.

This study was approved by the Institutional Review Board of SUNY Upstate Medical University, and all participants provided signed, informed consent in accordance with the Declaration of Helsinki.

The individuals who implemented the FS processing pipeline were blind to the diagnostic status of study participants.

### Imaging study

The 1.5T imaging data were acquired in the axial plane on a 1.5T Philips Interra scanner (Philips Medical Systems, Best, The Netherlands) utilizing the following T1-weighted inversion recovery, turbo gradient echo (TFE) 3-D pulse sequence: echo time = 4.6 ms; repetition time = 20 ms; 2 repetitions; matrix size 256 × 154; field of view = 24 cm; multishot = 32; TFE pre-inversion recovery = 394 ms, 1.5 mm slice thickness (Kates et al., 2011b).

The 3T imaging data were acquired in the sagittal plane on a 3T Siemens Magnetom Trio Tim scanner (syngo MR B17, Siemens Medical Solutions, Erlangen, Germany) utilizing an ultrafast gradient echo 3D sequence (MPRAGE) with PAT k-space-based algorithm GRAPPA and the following parameters: echo time = 3.31 ms; repetition time = 2530 ms; matrix size 256 × 256; field of view = 256 mm, slice thickness = 1 mm.

### Image analysis

#### Imaging data preprocessing

Preprocessing of 1.5T imaging data consisted of generating an isotropic brain image with non-brain tissue removed, and aligning that image along the anterior-posterior commissure. This was accomplished by importing the raw 1.5T MRI images into the imaging software program, BrainImage (available from the Center for Interdisciplinary Brain Sciences Research, Stanford University), where we performed an initial intensity correction, an automatic brain mask creation, followed by a manual editing step of the brainmask (Subramaniam et al., 1997). After the final manual editing, the skull was removed from the image and the brain image was saved in Analyze file format for import into the imaging software package, 3DSlicer (www.slicer.org; Fedorov et al., 2012). In 3DSlicer, the skull-stripped brains were aligned along the anterior and posterior commissure axis, and then re-sampled into isotropic voxels (0.9375 mm^3^) using a cubic spline interpolation transformation.

Preproccessing of 3T images also consisted of generating an isotropic brain image with non-brain tissue removed. However, instead of using BrainImage to remove non-brain tissue, we used the initial, preprocessing step in the FS pipeline. The resulting brain mask was imported into 3DSlicer, and manually edited using the same steps included in the protocol cited above. Afterwards, the skull was removed from the image and the brain image was aligned along the anterior and posterior commissure axis using a cubic spline transformation and kept at the same resolution as the initial data, isotropic voxels (1 mm^3^).

At that point, both 1.5T and 3T edited and aligned brain masks were subject to the FreeSurfer segmentation process, described below.

#### FS segmentation process

The preprocessed images were imported into the automated brain segmentation software FreeSurfer (FS) installed on a Dell Optiplex machine using the Ubuntu 12.04 operating system. In addition to resampling of the image into 0.9375 mm^3^ using a cubic spline transformation during preprocessing as described above, the FS segmentation process resampled the images into 1 mm^3^ as part of its motion correction step. Cortical reconstruction and volumetric segmentation was performed with the Freesurfer image analysis suite, which is documented and freely available for download online (http://surfer.nmr.mgh.harvard.edu/). The technical details of these procedures are described in prior publications (Dale and Sereno, 1993; Dale et al., 1999; Fischl et al., 1999a,b, 2001, 2002, 2004a,b; Fischl and Dale, 2000; Ségonne et al., 2004; Han et al., 2006; Jovicich et al., 2006).

Briefly, the FS segmentation process included: the segmentation of the subcortical white matter and deep gray matter volumetric structures (including hippocampus, amygdala, caudate, putamen, ventricles) (Fischl et al., 2002, 2004a); intensity normalization (Sled et al., 1998); tessellation of the gray matter white matter boundary; automated topology correction (Fischl et al., 2001; Ségonne et al., 2007); and surface deformation following intensity gradients to optimally place the gray/white and gray/cerebrospinal fluid borders at the location where the greatest shift in intensity defines the transition to the other tissue class (Dale and Sereno, 1993; Dale et al., 1999; Fischl and Dale, 2000). Once the cortical models were complete, a number of deformable procedures were performed including surface inflation (Fischl et al., 1999a), registration to a spherical atlas which utilizes individual cortical folding patterns to match cortical geometry across subjects (Fischl et al., 1999b), parcellation of the cerebral cortex into units based on gyral and sulcal structure (Fischl et al., 2004b; Desikan et al., 2006), and creation of a variety of surface based data including maps of curvature and sulcal depth. Details of the methods involved have been described extensively elsewhere (Fischl and Dale, 2000; Salat et al., 2004).

#### Final steps of fully automated (unedited) pipeline

Following the successful completion of the FS reconstruction process, the FS directories were duplicated, and one copy immediately underwent the final reconstruction stream without manual intervention. Cortical thickness, surface area and white matter volume measurements were extracted for selected Region of Interest (ROIs) and the directories were backed up to a remote and secure location. Cortical thickness measurements were computed by looking at the average distance, calculated using a spatial lookup table, between the white matter and pial surfaces generated by FS (Fischl and Dale, 2000). This group of FS data without any manual intervention will be referred to as “unedited.”

#### Final steps of manual intervention (edited) method

The second copy of the data were manually inspected for defects that could affect the accuracy of the final cortical measurements. The full protocols for processing and editing both 1.5T and 3T data are provided in Supplementary Material; however a brief description of the process follows. In the coronal view, starting posteriorly, with the opposite hemisphere of the brain obstructed in order to minimize human error, each slice was inspected for errors in the surfaces created by FS. An error can be described as an instance where one of the surfaces drawn by FS includes or excludes voxels incorrectly. These errors are most often caused by motion artifacts in the more posterior sections of the brain, and by hyperintensities around the temporal and orbitofrontal lobes. Control Points, manually inserted targets that adjust a voxel's intensity value to 110, were inserted within adjacent white matter regions in order to correct surface errors as described on the FS website^2^. Where appropriate, hyperintensities, and extraneous tissue were removed from the brain volume as well, as described in the White Matter Edits tutorial on the FS website^3^. Once completed, the process was repeated for the opposite hemisphere. After all errors were corrected, the brain was re-run through the second reconstruction stream beginning at the module where control point adjusted voxels are taken into account. This process was repeated up to four times to ensure all errors in FS surfaces were corrected.

Following successful correction of the FS surfaces, the final reconstruction step was run and cortical thickness and volume measurements were extracted for all ROIs. Manually-corrected data, hereafter referred to as “edited,” were then compared with the unedited data.

### Statistical analyses

Analyses comparing the unedited and edited volumes and cortical thickness values for each ROI were run separately in SPSS (v22) for the 1.5T and 3T data. Accordingly, for both the 1.5T and the 3T data, the variance was calculated for each ROI, based on the total sample of 30 individuals, and the Levene's test was used to compare the variance of each edited ROI to that of each unedited ROI. Intraclass correlation coefficients between edited and unedited ROIs were calculated based on the total sample as well, and paired *t*-tests were conducted in order to determine if the means differed significantly between edited and unedited ROIs. The Bonferroni correction was applied to the 34 paired *t*-tests that we performed for each set of measures (i.e., surface area, white matter volume, thickness) at each field strength.

As noted above, we also generated effect sizes for the mean surface areas/white matter volumes/cortical thickness values between the 20 individuals with 22q11DS and the 10 controls, in order to determine the differences in effect sizes that the edited vs. unedited methods yielded. This would allow one to determine the sample sizes for edited vs. unedited methods that would be necessary to detect significant differences in volume/cortical thickness between individuals with 22q11DS and controls. To determine whether effect sizes for the edited method differed significantly from effect sizes for the unedited method, we calculated paired *t*-tests across all ROIs. Bonferroni corrections were applied to paired *t*-tests as described above. In addition, we calculated the arithmetic difference in effect size for each edited vs. unedited ROI (by subtracting the unedited value from the edited value).

## Results

**Figure 1:**
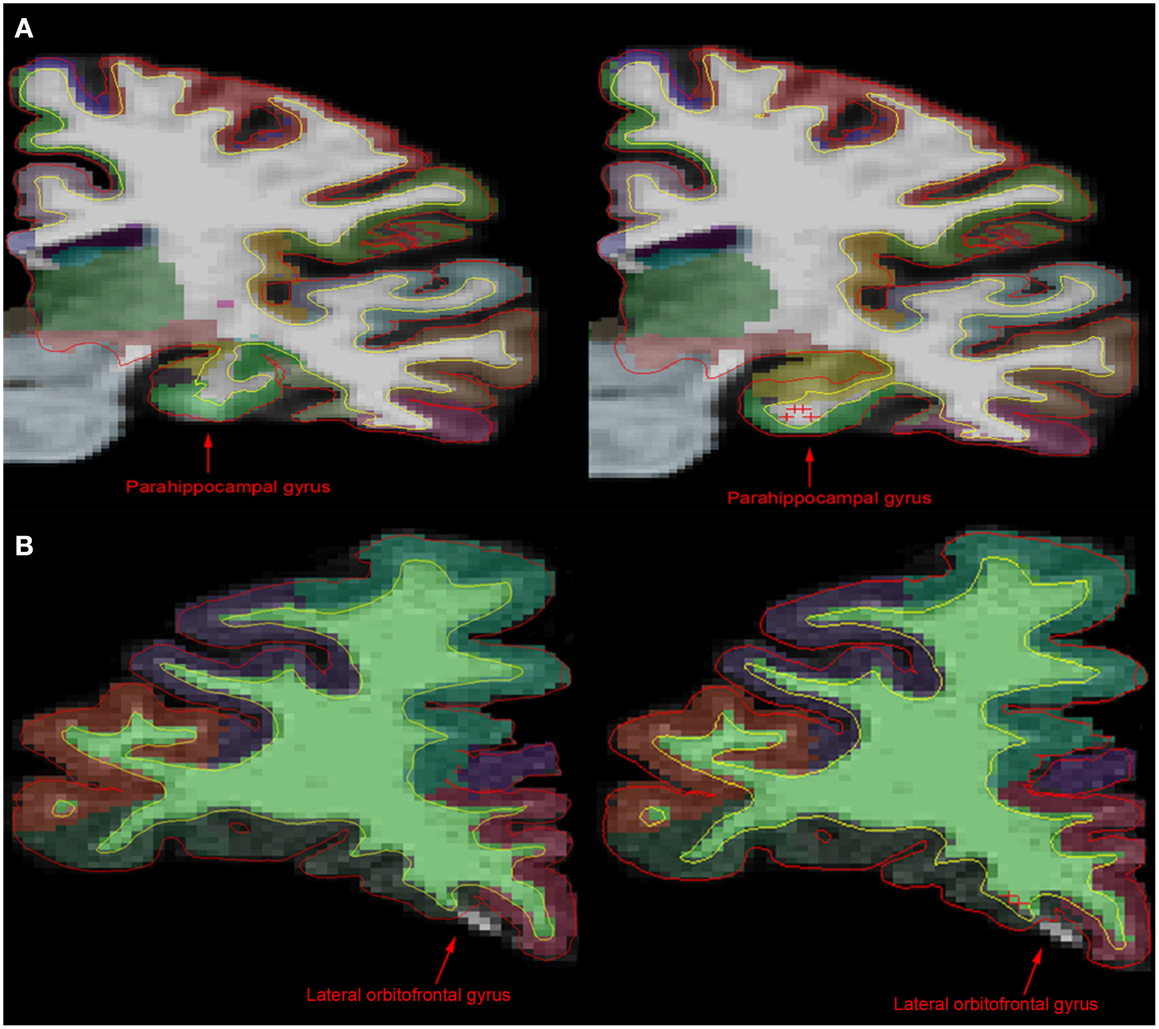
**Comparison of MR images before and after manual intervention**. **(A)** In comparison with the unedited 1.5T image (left), the manually edited brain image (right) shows a more accurate portrayal of the parahippocampal gyrus, the hippocampus and the white matter boundary. **(B)** However, in the 3T brain images, there is little difference between the unedited (left) and the manually edited (right) images. The manual intervention implemented in the 3T brain was intended to include white matter and gray matter incorrectly being excluded from the lateral orbitofrontal gyrus area. Control points on this slice in addition to edits on anterior and posterior brain slices had no significant effect on the exclusion. This shows that although control points can have an effect on white matter and pial surface, as well as cortical parcellation, it is inconsistent.

**Figure 2:**
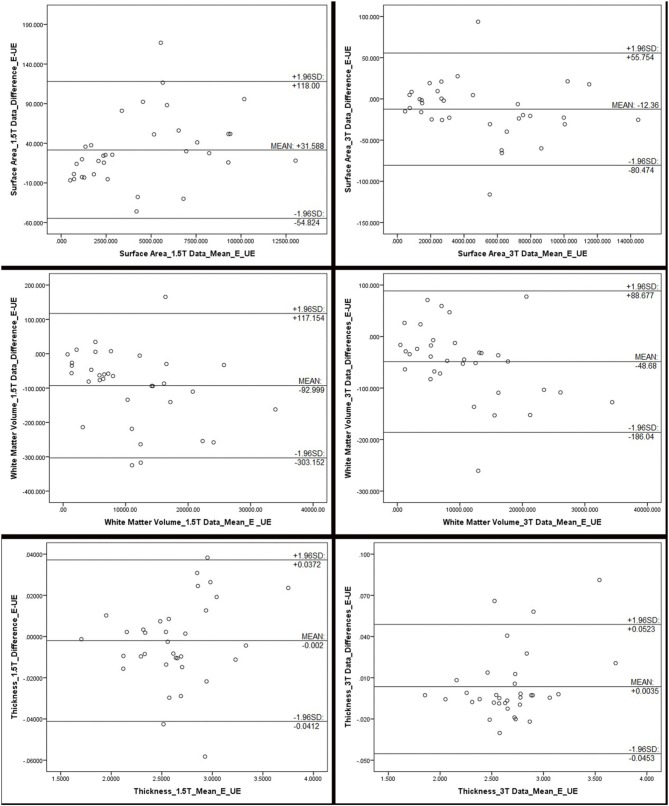
**Bland Altman plots, representing the differences between edited and unedited measures of surface area, white matter volume and cortical thickness for each field strength**. The difference between the edited and unedited measure of each region of interest is plotted against the average of the two measures. Mean, and 95% limits, of agreement are provided in each plot. These plots indicate that, for the most part, the two methods are producing somewhat similar results, although all plots show a fairly wide range of values. Outliers, beyond the 95% agreement limit, indicating poor agreement, include: for surface area (1.5T): inferior temporal gyrus; surface area (3T): lateral orbitofrontal gyrus and insula; white matter volume (1.5T): insula, fusiform gyrus, inferior temporal gyrus; white matter volumes (3T): lateral orbitofrontal gyrus; thickness (1.5T): rostral anterior cingulate, pars orbitalis, and parahippocampal gyrus; thickness (3T): entorhinal cortex, inferior temporal gyrus, and medial orbitofrontal gyrus.

**Figure 3:**
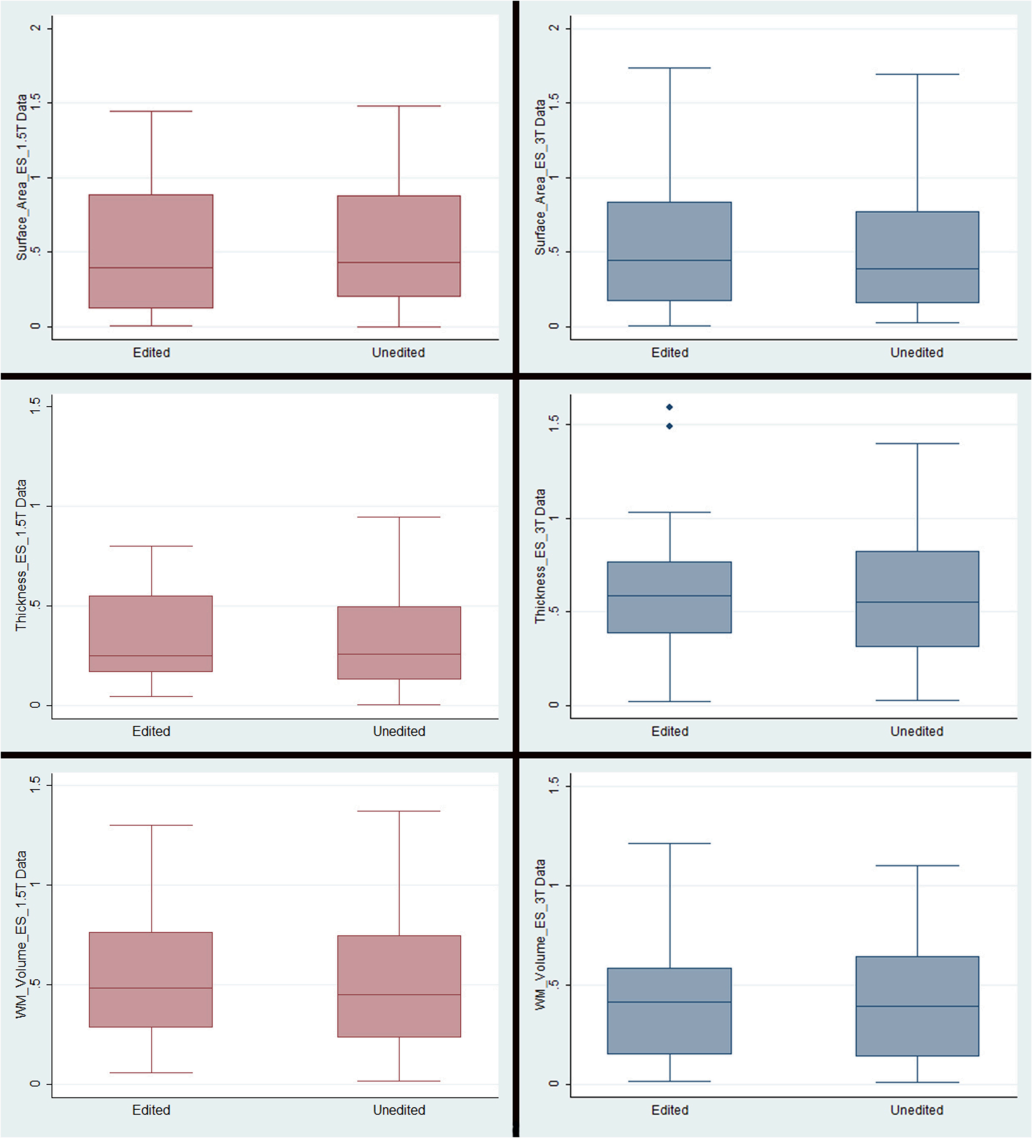
**Box plots representing means and standard deviations of effect sizes for each measurement type/field strength**. Note that the only outliers were in the cortical thickness plots for the 3T data. The outlying regions of interest were pericalcarine thickness (1.49) and medial orbitofrontal thickness (1.60).

Figure 1 compares MR images with and without manual intervention with control points. Means and standard deviations for surface area, white matter volume, and cortical thickness for each ROI, separated by scanner field strength, are provided in Table 2. The differences between edited and unedited measures are represented by Bland—Altman plots in Figure 2. Variances and intraclass correlation coefficients for all ROIs, separated by scanner field strength, are provided in Table 3. Effect sizes are provided in Table 4 and box plots representing effect sizes are provided in Figure 3.

**Table 2:** **Means and standard deviations of scanner-specific surface area, volume and cortical thickness values for FreeSurfer regions of interest**.

**Region**		**Surface area mm^3^**		**White Matter volume mm^3^**		**Cortical thickness mm**	
		**1.5T**	**3T**	**1.5T**	**3T**	**1.5T**	**3T**
Banks of STS^a^	E	1819.23±303.37	2045.43±243.57	5182.13±953.37	5260.23±796.00	2.57±0.19	2.71±0.14
	UE	1820.07±316.80	2070.33±247.34	5147.77±1028.18	5342.93±793.98	2.56±0.19	2.73±0.12
Caudal anterior cingulate	E	1160.30±181.70	1337.60±205.74	5169.43±834.40	5311.00±714.43	2.54±0.30	2.56±0.16
	UE	1157.63±193.51	1338.00±207.51	5163.97±844.28	5349.87±722.07	2.54±0.30	2.59±0.17
Caudal middle frontal	E	4272.73±545.80	4538.07±585.50	12,247.07±1415.65	12,490.77±1817.58	2.67±0.18	2.72±0.11
	UE	4244.93±561.78	4533.43±589.27	12,252.60±1486.97	12,542.23±1887.66	2.70±0.19	2.74±0.11
Cuneus	E	2368.60±367.26	2652.97±391.10	4050.53±848.29	3713.60±709.58	1.96±0.14	2.05±0.11
	UE	2384.13±392.15	2631.97±383.92	4131.67±885.20	3689.90±661.60	1.95±0.15	2.06±0.10
Entorhinal	E	708.10±177.26	733.10±178.68	1323.03±409.72	1179.90±316.51	3.33±0.27	3.58±0.32
	UE	709.27±148.46	728.30±144.38	1379.70±411.88	1243.57±280.04	3.33±0.30	3.50±0.33
Fusiform	E	5591.43±794.38	6236.23±695.59	12,265.73±1951.86	12,191.40±1838.86	2.61±0.15	2.85±0.13
	UE	5707.93±738.88	6301.90±725.68	12,583.10±1979.50	12,327.93±1879.31	2.62±0.15	2.82±0.13
Inferior parietal	E	9276.30±1158.45	10,223.63±1139.99	20,702.47±2720.77	20,689.87±2833.43	2.49±0.14	2.65±0.12
	UE	9328.00±1167.95	10,202.30±1116.62	20,812.97±2839.40	20,612.40±2767.54	2.48±0.14	2.65±0.11
Inferior temporal	E	5457.40±895.73	6223.77±787.19	10,858.73±2046.67	10,668.07±1800.12	2.87±0.14	2.93±0.16
	UE	5624.20±898.59	6286.13±789.98	11,183.50±2222.92	10,712.70±1844.55	2.83±0.14	2.87±0.15
Isthmus cingulate	E	1648.90±235.37	1946.03±295.78	6375.73±873.63	7105.00±1092.52	2.63±0.15	2.47±0.14
	UE	1686.40±244.98	1926.90±273.27	6448.93±925.17	7045.73±1060.07	2.64±0.16	2.45±0.14
Lateral occipital	E	8214.50±922.15	8606.73±946.60	17,090.40±2420.78	15,477.23±2254.65	2.32±0.18	2.31±0.10
	UE	8242.03±922.87	8666.70±933.38	17,231.17±2384.35	15,630.27±2267.58	2.32±0.18	2.31±0.09
Lateral orbitofrontal	E	4502.70±496.50	5485.20±444.89	12,252.67±1479.24	12,805.97±1367.90	2.94±0.15	2.67±0.11
	UE	4595.07±473.11	5601.20±506.88	12,516.47±1492.90	13,066.60±1494.30	2.93±0.16	2.63±0.12
Lingual	E	5138.27±675.92	5541.47±723.15	10,252.07±1197.55	9206.50±1279.54	2.11±0.12	2.16±0.12
	UE	5189.47±677.79	5572.20±742.89	10,386.10±1291.08	9219.00±1220.67	2.13±0.16	2.16±0.10
Medial orbitofrontal	E	3333.27±562.31	3632.47±411.20	7189.00±1221.98	5881.97±1038.60	2.87±0.25	2.56±0.10
	UE	3414.27±460.99	3604.97±363.91	7247.13±1051.96	5949.70±1001.70	2.84±0.23	2.49±0.08
Middle temporal	E	5841.03±757.97	6544.97±770.48	10,884.57±1837.63	10,453.53±1737.59	2.99±0.14	3.06±0.14
	UE	5929.13±824.99	6584.70±759.82	11,103.37±1964.15	10,506.30±1719.80	2.97±0.15	3.06±0.14
Parahippocampal	E	1334.03±221.01	1456.33±159.73	3032.67±548.63	3146.27±461.45	2.50±0.38	2.73±0.26
	UE	1369.73±204.17	1457.90±151.42	3246.57±492.53	3170.23±523.92	2.54±0.37	2.72±0.25
Paracentral	E	2587.87±348.16	2779.23±345.32	7662.50±1225.01	7949.00±1139.90	2.29±0.17	2.54±0.11
	UE	2582.53±359.13	2781.33±350.27	7655.17±1248.13	7996.13±1173.09	2.30±0.16	2.54±0.11
Pars opercularis	E	2831.67±287.14	3104.43±317.99	6544.37±826.25	6801.97±985.12	2.69±0.18	2.77±0.14
	UE	2857.20±268.48	3127.27±334.22	6604.57±799.93	6873.30±1007.83	2.71±0.19	2.78±0.14
Pars orbitalis	E	1281.47±138.83	1420.77±158.52	2149.93±360.75	1950.20±365.86	2.97±0.21	2.78±0.15
	UE	1278.23±159.80	1436.83±146.09	2138.30±411.71	1984.50±341.42	2.93±0.26	2.78±0.17
Pars triangularis	E	2452.63±332.56	2649.00±327.66	5821.23±862.96	5727.13±720.66	2.73±0.15	2.63±0.12
	UE	2477.87±355.90	2648.60±311.20	5898.23±954.73	5734.17±732.89	2.73±0.14	2.63±0.12
Pericalcarine	E	2360.67±368.73	2665.93±438.50	5781.17±1016.05	5372.37±1010.50	1.70±0.17	1.85±0.12
	UE	2336.37±369.70	2691.53±435.63	5844.13±1026.37	5389.70±958.03	1.71±0.18	1.86±0.11
Post central	E	7541.93±990.56	7960.33±988.90	14,304.80±2303.64	13,430.83±2173.77	2.12±0.15	2.26±0.11
	UE	7583.07±1017.59	7980.97±996.23	14,398.90±2356.20	13,462.90±2147.87	2.13±0.14	2.26±0.12
Posterior cingulate	E	2063.97±256.27	2418.10±236.91	7950.73±928.77	8365.77±933.22	2.54±0.18	2.57±0.12
	UE	2081.57±254.15	2408.67±233.94	8015.83±979.65	8318.60±909.87	2.55±0.18	2.58±0.12
Precentral	E	9286.70±774.28	10,031.37±761.91	25,719.97±2699.72	26,046.97±2767.75	2.56±0.15	2.65±0.10
	UE	9302.53±797.96	10,062.20±788.57	25,752.80±2766.48	26,155.40±2854.26	2.59±0.14	2.66±0.11
Precuneus	E	6504.47±734.21	7222.87±783.66	16,107.43±2516.59	16,115.53±2422.52	2.33±0.13	2.57±0.11
	UE	6560.60±746.79	7229.23±767.92	16,194.27±2559.96	16,224.70±2379.18	2.36±0.13	2.57±.11
Rostral anterior cingulate	E	1149.37±202.39	1484.23±173.50	4480.97±603.32	4851.10±657.92	2.90±0.24	2.88±0.16
	UE	1169.20±213.35	1489.37±189.74	4527.80±628.02	4780.20±712.05	2.96±0.25	2.88±0.17
Rostral middle frontal	E	10,131.67±1280.86	11,517.80±1413.66	23,941.70±3363.86	23,406.83±3120.46	2.56±0.18	2.47±0.10
	UE	10,227.37±1335.90	11,500.07±1399.77	24,199.93±3441.36	23,510.23±3120.11	2.56±0.18	2.49±0.10
Superior frontal	E	13,023.13±1513.54	14,437.67±1523.73	33,892.20±4741.96	34,307.17±4872.37	2.93±0.16	2.86±0.11
	UE	13,041.17±1500.01	14,463.00±1575.10	34,054.40±4744.88	34,434.57±5075.83	2.95±0.18	2.88±0.12
Superior parietal	E	9379.53±853.69	9976.73±884.97	22,198.90±2452.70	21,167.63±2423.51	2.16±0.13	2.38±0.11
	UE	9431.37±875.09	9999.43±880.61	22,453.13±2550.07	21,320.03±2462.00	2.15±0.14	2.39±0.10
Superior temporal	E	6814.20±487.93	7278.53±644.35	14,132.53±1432.51	13,095.77±1897.51	2.69±0.22	2.89±0.11
	UE	6784.20±577.45	7302.23±622.04	14,226.50±1468.14	13,127.07±1871.21	2.70±0.22	2.90±0.11
Supramarginal	E	6940.83±806.34	7530.60±836.41	16,515.77±2261.22	16,121.80±2337.31	2.65±0.16	2.77±0.12
	UE	6970.70±872.17	7550.27±830.43	16,545.70±2380.54	16,158.20±2393.48	2.66±0.15	2.78±0.12
Frontal pole	E	509.47±70.64	456.40±58.98	707.27±114.40	480.23±96.73	3.05±0.34	2.73±0.23
	UE	502.73±77.85	471.47±61.81	709.10±130.69	496.57±92.22	3.03±0.31	2.72±0.24
Temporal pole	E	850.30±93.61	858.40±97.08	1375.10±170.90	115.87±199.39	3.76±0.27	3.71±0.31
	UE	864.30±96.36	849.87±106.56	1409.97±179.53	1130.40±186.80	3.74±0.24	3.69±0.29
Transverse temporal	E	720.70±108.68	738.47±119.25	1395.90±190.35	13,210.47±227.56	2.34±0.22	2.52±0.12
	UE	715.53±105.73	749.53±122.30	1422.40±211.11	1349.20±204.54	2.33±0.21	2.52±0.13
Insula	E	4215.53±363.26	4890.70±533.42	16,456.60±1570.42	17,657.50±1600.45	3.22±0.19	3.14±0.14
	UE	4169.50±394.89	4797.03±444.90	16,291.13±1705.30	17,705.93±1694.21	3.24±0.17	3.15±0.14

a Banks of the superior temporal sulcus.

**Table 3:** **Variances and intraclass correlation coefficients (ICC), based on comparisons of “edited” and “unedited” processing pipelines measuring surface area, white matter volume and cortical thickness**.

**Region**	**Surface area**				**White matter volume**				**Cortical thickness**			
	**Variance**		**ICC**		**Variance**		**ICC**		**Variance**		**ICC**	
	**1.5T**	**3T**	**1.5T**	**3T**	**1.5T**	**3T**	**1.5T**	**3T**	**1.5T**	**3T**	**1.5T**	**3T**
Banks of STS^a^	0.115	0.008	0.926	0.959	0.137	0.012	0.967	0.970	0.018	0.064	0.958	0.967
Caudal anterior cingulate	0.073	0.001	0.983	0.988	0.002	0.081	0.983	0.971	0.006	0.001	0.933	0.922
Caudal middle frontal	0.000	0.026	0.971	0.987	0.159	0.113	0.957	0.987	0.003	0.013	0.953	0.938
Cuneus	0.105	0.008	0.982	0.984	0.018	0.003	0.979	0.982	0.028	0.030	0.890	0.962
Entorhinal	0.442	1.522	0.521	0.856	0.153	0.634	0.597	0.879	0.594	0.091	0.813	0.917
Fusiform	0.549	0.068	0.930	0.986	0.002	0.023	0.950	0.989	0.022	0.190	0.875	0.942
Inferior parietal	0.009	0.017	0.984	0.996	0.029	0.005	0.979	0.995	0.034	0.000	0.924	0.974
Inferior temporal	0.000	0.002	0.924	0.988	0.115	0.004	0.946	0.994	0.055	0.002	0.758	0.892
Isthmus cingulate	0.296	0.040	0.821	0.960	0.054	0.022	0.923	0.980	0.037	0.020	0.893	0.910
Lateral occipital	0.002	0.032	0.975	0.988	0.070	0.005	0.972	0.987	0.005	0.294	0.985	0.970
Lateral orbitofrontal	0.041	0.516	0.920	0.894	0.020	0.084	0.948	0.935	0.361	0.553	0.879	0.858
Lingual	0.002	0.001	0.958	0.984	0.244	0.054	0.915	0.973	0.627	0.416	0.898	0.920
Medial orbitofrontal	1.106	0.049	0.902	0.868	0.496	0.046	0.901	0.915	0.030	0.668	0.892	0.646
Middle temporal	0.219	0.102	0.951	0.989	0.152	0.035	0.963	0.989	0.018	0.108	0.798	0.982
Parahippocampal	0.002	0.144	0.208	0.888	0.012	0.338	0.343	0.936	0.043	0.036	0.952	0.980
Paracentral	0.015	0.007	0.992	0.993	0.025	0.027	0.985	0.985	0.135	0.014	0.914	0.955
Pars opercularis	0.205	0.147	0.899	0.967	0.145	0.017	0.922	0.955	0.100	0.052	0.946	0.972
Pars orbitalis	0.138	0.550	0.880	0.969	0.130	0.113	0.933	0.981	0.303	0.355	0.844	0.980
Pars triangularis	0.086	0.208	0.940	0.967	0.009	0.005	0.930	0.968	0.279	0.039	0.910	0.959
Pericalcarine	0.000	0.002	0.972	0.979	0.014	0.000	0.973	0.983	0.091	0.008	0.968	0.941
Post central	0.021	0.003	0.993	0.996	0.018	0.018	0.989	0.992	0.099	0.009	0.982	0.986
Posterior cingulate	0.039	0.000	0.984	0.988	0.202	0.167	0.965	0.969	0.037	0.013	0.857	0.974
Precentral	0.001	0.094	0.988	0.994	0.006	0.068	0.984	0.994	0.053	0.002	0.945	0.972
Precuneus	0.005	0.002	0.986	0.997	0.011	0.011	0.991	0.995	0.004	0.010	0.942	0.974
Rostral anterior cingulate	0.032	0.612	0.949	0.903	0.000	0.395	0.951	0.938	0.029	0.048	0.927	0.893
Rostral middle frontal	0.086	0.013	0.990	0.993	0.045	0.014	0.989	0.990	0.220	0.052	0.966	0.938
Superior Frontal	0.009	0.021	0.993	0.996	0.015	0.011	0.990	0.997	0.265	0.052	0.941	0.936
Superior parietal	0.000	0.000	0.988	0.993	0.066	0.011	0.983	0.992	0.268	0.000	0.961	0.979
Superior temporal	0.172	0.074	0.898	0.981	0.007	0.011	0.956	0.986	0.020	0.000	0.976	0.984
Supramarginal	0.245	0.003	0.983	0.994	0.086	0.027	0.988	0.993	0.187	0.028	0.937	0.982
Frontal pole	0.161	0.029	0.886	0.883	0.230	0.141	0.859	0.921	0.131	0.000	0.903	0.873
Temporal pole	0.071	0.004	0.822	0.862	0.130	0.865	0.853	0.829	0.985	0.148	0.794	0.919
Transverse temporal	0.078	0.001	0.935	0.973	0.412	0.172	0.855	0.898	0.284	0.679	0.940	0.930
Insula	0.259	1.582	0.833	0.799	0.087	0.019	0.879	0.923	1.198	0.033	0.925	0.810

Data from both 1.5 tesla (1.5 T) and 3 tesla (3T) scanners are provided.

a Banks of the superior temporal sulcus.

**Table 4:** **Effect sizes (Cohen's d) based on comparisons of means of surface area, white matter volumes and cortical thickness, between individuals with 22q11.2 deletion syndrome (*N* = 20) and typical controls (*N* = 10)**.

**Region**		**Surface area**		**White matter volume**		**Cortical thickness**	
		**1.5T**	**3T**	**1.5T**	**3T**	**1.5T**	**3T**
Banks of STS^a^	E	0.371	0.047	0.182	0.016	0.785	0.025
	UE	0.279	0.246	0.235	0.150	0.813	0.167
caudal anterior cingulate	E	0.726	0.942	0.720	0.585	0.551	0.206
	UE	0.765	1.070	0.744	0.703	0.117	0.142
Caudal middle frontal	E	0.125	0.066	0.200	0.290	0.165	0.596
	UE	0.273	0.030	0.024	0.260	0.298	0.780
Cuneus	E	1.404	1.660	1.229	1.021	0.449	0.740
	UE	1.284	1.677	1.303	0.999	0.357	0.823
Entorhinal	E	0.095	0.398	0.463	0.546	0.190	0.692
	UE	0.358	0.191	0.189	0.492	0.141	0.623
Fusiform	E	0.889	0.533	1.007	0.391	0.383	0.061
	UE	0.695	0.497	1.076	0.402	0.315	0.025
Inferior parietal	E	0.018	0.342	0.107	0.539	0.047	0.271
	UE	0.069	0.410	0.014	0.613	0.274	0.308
Inferior temporal	E	0.199	0.276	0.473	0.123	0.395	0.574
	UE	0.210	0.277	0.415	0.114	0.004	0.521
Isthmus cingulate	E	0.119	0.488	0.437	0.791	0.160	0.714
	UE	0.249	0.362	0.358	0.805	0.089	0.558
Lateral occipital	E	0.953	0.629	0.932	0.341	0.135	0.201
	UE	0.978	0.542	0.816	0.277	0.124	0.305
Lateral orbitofrontal	E	0.202	0.017	0.057	0.361	0.212	0.621
	UE	0.021	0.185	0.157	0.456	0.050	0.395
Lingual	E	1.403	1.355	0.961	0.787	0.314	0.444
	UE	1.180	1.333	1.101	0.681	0.456	0.884
Medial orbitofrontal	E	0.113	0.008	0.203	0.152	0.250	1.596
	UE	0.107	0.066	0.195	0.278	0.174	1.402
Middle temporal	E	0.242	0.083	0.321	0.053	0.177	0.486
	UE	0.197	0.039	0.369	0.064	0.318	0.532
Parahippocampal	E	0.005	0.054	0.547	0.330	0.768	0.578
	UE	0.002	0.153	0.527	0.515	0.646	0.532
Paracentral	E	0.791	0.675	0.571	0.151	0.218	1.004
	UE	0.815	0.578	0.612	0.057	0.224	0.957
Pars opercularis	E	0.257	0.837	0.597	1.213	0.486	1.011
	UE	0.437	0.752	0.403	1.099	0.498	1.028
Pars orbitalis	E	0.020	0.331	0.155	0.474	0.196	0.762
	UE	0.285	0.203	0.265	0.399	0.241	0.701
Pars triangularis	E	0.888	0.867	0.713	0.087	0.122	0.802
	UE	1.101	0.834	0.469	0.008	0.147	0.799
Pericalcarine	E	0.841	1.395	0.583	0.718	0.162	1.493
	UE	0.876	1.687	0.520	0.739	0.216	1.371
Post central	E	0.847	0.848	0.764	0.216	0.234	0.967
	UE	0.872	0.820	0.701	0.153	0.300	0.956
Posterior cingulate	E	0.548	0.542	0.284	0.026	0.398	0.069
	UE	0.590	0.549	0.136	0.108	0.597	0.159
Precentral	E	0.006	0.185	0.172	0.459	0.586	0.887
	UE	0.021	0.186	0.186	0.385	0.731	0.819
Precuneus	E	1.418	1.277	1.252	0.598	0.183	0.650
	UE	1.386	1.250	1.244	0.564	0.127	0.699
Rostral anterior cingulate	E	0.016	0.337	0.396	0.586	0.636	0.507
	UE	0.048	0.075	0.285	0.648	0.603	0.263
Rostral middle frontal	E	1.446	1.736	1.300	0.919	0.070	0.702
	UE	1.479	1.691	1.374	0.855	0.146	0.720
Superior frontal	E	0.535	0.148	0.485	0.022	0.637	0.384
	UE	0.481	0.171	0.567	0.022	0.429	0.471
Superior parietal	E	1.231	0.822	1.038	0.270	0.125	0.487
	UE	1.282	0.770	1.063	0.241	0.016	0.451
Superior temporal	E	0.630	0.172	0.576	0.131	0.727	0.051
	UE	0.549	0.105	0.683	0.135	0.526	0.044
Supramarginal	E	0.425	0.042	0.484	0.085	0.580	1.036
	UE	0.425	0.054	0.504	0.123	0.693	1.206
Frontal pole	E	0.064	0.322	0.215	0.488	0.115	0.540
	UE	0.081	0.137	0.429	0.124	0.131	0.545
Temporal pole	E	0.138	0.318	0.297	0.772	0.433	0.558
	UE	0.325	0.031	0.073	0.553	0.132	0.546
Transverse temporal	E	1.339	0.663	1.049	0.433	0.250	0.023
	UE	1.230	0.664	0.857	0.643	0.013	0.086
Insula	E	0.248	0.632	0.448	0.585	0.801	0.768
	UE	0.033	0.437	0.417	0.336	0.947	1.245

Effect sizes of comparisons are reported for edited (E) and unedited (UE) measures, derived from 1.5 tesla (1.5T) and 3 tesla (3T) scanners.

a Banks of the superior temporal sulcus.

### Philips 1.5T data

#### Surface area measures

Levene's test indicated that the variance of each edited region of interest did not differ significantly from its unedited counterpart. Intraclass correlation analyses between unedited and edited surface areas yielded coefficients ranging from 0.82 to 0.99 for 32 out of the 34 ROIs. The only exceptions were entorhinal cortex areas (0.52) and parahippocampal gyrus areas (0.21). After Bonferroni correction, paired *t*-tests indicated that mean areas did not differ significantly between any unedited and edited ROIs.

Paired *t*-tests indicated that the mean effect size for surface areas did not differ significantly from the mean effect size for unedited areas. Moreover, the mean arithmetic difference in effect size between all edited and unedited surface area ROIs was −0.011 (SD 0.12). The regions for which the difference in effect size between edited and unedited methods exceeded either 0.20 or −0.20 (indicating small effect sizes) for was the entorhinal cortex (−0.26), lingual area (0.22), pars orbitalis (−0.27), and pars triangularis (−0.21).

#### White matter volumes

No significant differences were observed in variances of white matter volumes between edited and unedited ROIs. Intraclass correlation analyses between unedited and edited white matter volumes yielded coefficients ranging from 0.85 to 0.99 for 32 out of 34 ROIs. Similar to surface areas, the exceptions were entorhinal cortex (0.60) and parahippocampal gyrus (0.34) volumes. Mean volumes did not differ significantly between 32 of the 34 pairs of unedited and edited regions. Exceptions were the lateral orbitofrontal (*p* < 0.001) cortex and the superior parietal lobule (*p* < 0.001).

The mean effect size for edited measures of white matter volumes did not differ significantly from the mean effect size for unedited measures. The mean arithmetic difference in effect size between all edited and unedited white matter ROIs was −0.018 (SD 0.11). The regions with the largest differences in effect sizes between edited and unedited methods for measuring white matter volumes were the entorhinal cortex (0.27), the pars triangularis (0.24), the frontal pole (−0.21) and the temporal pole (0.22).

#### Cortical thickness

No significant differences were observed in variances of cortical thickness between edited and unedited ROIs. Intraclass correlation analyses between unedited and edited measures of cortical thickness yielded coefficients ranging from 0.84 to 0.985 for 31 out of 34 ROIs. Exceptions included entorhinal cortex (0.81), inferior temporal gyrus (0.76) and the temporal pole (0.79). Mean cortical thickness did not differ significantly between 32 of the 34 pairs of unedited and edited regions. Exceptions were the precentral gyrus (*p* < 0.001) and the rostral anterior cingulate (*p* < 0.001).

The mean effect size for edited measures of cortical thickness did not differ significantly from the mean effect size for unedited measures. The mean arithmetic difference in effect size between all edited and unedited measures of cortical thickness was −0.03 (SD 0.16). The regions with the largest differences in effect size between edited and unedited methods were the caudal anterior cingulate (0.43), fusiform gyrus (−0.23), inferiorparietal lobule (0.39), rostral anterior cingulate (0.21), superior frontal gyrus (0.20), supramarginal gyrus (0.30) and temporal pole (0.24). Note that the majority of these values were positive, indicating that the effect sizes for the edited method tended to be larger than those for the unedited method used to measure cortical thickness.

### Siemens 3T data

#### Surface area measures

For the 3T data, Levene's test similarly indicated that the variance of each edited region of interest did not differ significantly from its unedited counterpart. Intraclass correlation analyses between unedited and edited surface areas yielded coefficients ranging from 0.86 to 0.99 for 33 out of 34 ROIs. Exceptions included the insula (0.799). Paired *t*-tests indicated that mean surface areas did not differ significantly between any pairs of unedited and edited regions. However, several regions tended to differ, including the fusiform gyrus (*p* = 0.002), the lateral orbitofrontal area (*p* = 0.003), and the inferior temporal lobe (*p* = 0.004).

For the 3T data, the mean effect sizes for edited and unedited measures of surface area did not differ. The mean arithmetic difference in effect size between edited and unedited surface area ROIs was −0.028 (SD 0.12). The regions with the largest differences in effect sizes between the edited and unedited methods were the entorhinal cortex (0.21), pericalcarine cortex (−0.29), the rostral anterior cingulate (0.26), and the temporal pole (0.287).

#### White matter volumes

No significant differences were observed in the variances of white matter volumes between edited and unedited ROIs. Intraclass correlation analyses between unedited and edited white matter volumes yielded coefficients ranging from 0.90 to 1.00 for all ROIs. After Bonferonni correction, the mean white matter volumes did not differ significantly between any pairs of unedited and edited regions, however the fusiform gyrus (*p* < 0.005) and the pars orbitalis (*p* < 0.005) approached significance.

The mean effect size for edited measures of white matter volume did not differ significantly from the mean effect size for unedited measures. The mean arithmetic difference in effect size between edited and unedited white matter ROIs was −0.013 (SD 0.11). The regions with the largest differences in effect size between the unedited and edited methods were the frontal pole (0.369), temporal pole (0.22), transverse temporal cortex (0.21) and insula (0.25).

#### Cortical thickness

No significant differences in the 3T data were observed in variances of cortical thickness between edited and unedited ROIs. Intraclass correlation analyses between unedited and edited measures of cortical thickness yielded coefficients ranging from 0.86 to 0.986 for 32 out of 34 ROIs. Exceptions included medial orbitofrontal cortex (0.65) and the insula (0.81). In contrast to 1.5T data, mean cortical thickness differed significantly between 7 of the 34 pairs of unedited and edited regions, including the banks of the superior temporal sulcus, entorhinal cortex, fusiform gyrus, inferior temporal gyrus, lateral orbitofrontal cortex, medial orbitofrontal cortex and rostral middle frontal cortex (all *p* < 0.001). Moreover, an additional 3 ROIs approached significance, including the superior frontal gyrus (*p* < 0.003), precentralgyrus (*p* < 0.004) and the caudal middle frontal gyrus (*p* < 0.004).

The mean effect size for edited measures of cortical thickness did not differ significantly from the mean effect size for unedited measures. The mean arithmetic difference in effect size between edited and unedited measures of cortical thickness was 0.07 (SD 0.15). The regions with the largest differences in effect sizes were the lateral orbitofrontal cortex (0.226), the lingual gyrus (−0.439), the rostral anterior cingulate (0.244) and the insula (−0.47).

## Discussion

In the last 5 years, FreeSurfer (FS) has become the standard for obtaining cortical metrics from MRI images due to its ease of configuration, accurate results, and high reproducibility (Fischl et al., 2002; Tae et al., 2008; Bhojraj et al., 2011). However, there has been a lack of consensus around whether or not additional manual editing is required in order to increase the ability to detect effects between groups. This is the first study, to the best of our knowledge, to directly compare FS's fully automated method to that of FS's semi-automated manual intervention method that utilizes control points to alter gray-white matter boundaries. Overall we found very few differences between methodological approaches, although we do note specific exceptions below.

### 1.5T data

We found few differences between methodological approaches when using the FS segmentation process to obtain surface areas from 1.5T images. The absence of differences in variance, and the high level of intraclass correlation coefficients between the regions in edited and unedited brains support previous studies that have established the consistency and reproducibility of the fully automated FS segmentation process (Fischl et al., 2002). As found in previous studies, the regions where differences were observed, i.e., the entorhinal cortex and parahippocampal gyrus, are common locations for imaging artifacts (Oguz et al., 2008; Desikan et al., 2010). These results support previous research into FS's difficulty obtaining measurements in similar scenarios, rather than suggesting a difference between the two methods (Desikan et al., 2010). This is supported by an absence of significant differences in the mean volumes and mean effect sizes between the two methods for measuring surface areas.

Although some differences were observed in white matter volume variance, the absence of consistently larger effect sizes for either method further indicates that the differences should not be viewed as a higher level of accuracy in volume segmentation for either method. One exception may be the lateral orbitofrontal cortex, for which we observed significant differences in mean volume. Due to motion which causes commonly-occurring imaging artifacts, the lateral orbitofrontal cortex is a region where raters make numerous corrections (i.e., using control points) during the FS pipeline. Although in our data, the difference in effect size between our patient and control samples was negligible for this region, that may not be the case for other populations and therefore automated white matter volumes derived for this region in general, when using a 1.5T scanner, should be viewed with caution.

As described in the methods section, cortical thickness is derived from the distance between the white matter surface, which follows the border between white and gray matter, and the pial surface, which follows the border between gray matter and cerebrospinal fluid. Since manually inserting control points affects where those surfaces are positioned, the differences between the methods should be most pronounced in cortical thickness measurements. Although there was an absence of difference in the variance, ICC's, and mean cortical thickness for most regions, the difference in effect sizes was surprising. The caudal anterior cingulate, superior frontal gyrus, supramarginal gyrus, and temporal pole all had effect sizes which favored the edited method, but do not typically require many control points. On the other hand, the region that favored the unedited method, the fusiform gyrus, usually needs heavier manual correction to exclude hyper intensities. Although further exploration is needed in order to determine what specifically caused the unexpected results, it is possible that errors in the automated segmentation are more pronounced in 22q11DS due to enlarged ventricles, and that fusiform gyrus matter was incorrectly excluded in the unedited brains, giving the appearance of a larger effect then was actually present. Nonetheless, the lack of consistently significant differences in variances and mean cortical thickness volumes between the edited methods further supports the notion that manual intervention for 1.5T images in FS's automated process does not provide an increase in ability to detect an effect size between groups commensurate with the human hours required.

### 3T data

The results for surface area and white matter volume in 3T data are similar to what was observed for the 1.5T data, and suggest that consistency in method is most likely more important than the choice between the fully automated and the manual-edit procedures. This is corroborated by similar effect sizes observed for both the manual and automated process, with the exception of temporal and occipital lobe structures affected by the issues described above.

Although no significant differences were observed in cortical thickness variance between the two groups, a notable difference in the results between the 1.5T and 3T data were 7 regions with differences in mean cortical thickness. The relatively large number of regions in the 3T for which we observed differences, and the fact that the same differences weren't present in 1.5T data warrant further explanation. In particular, the superior temporal sulcus, and the lateral and medial orbitofrontal cortices typically require manual editing in both the 1.5T and 3T data.

It is possible that due to the higher contrast in 3T scans, the control points had greater success in correcting misplaced surfaces than in the 1.5 scans, potentially resulting in more accurate surfaces and cortical thickness measurements. This would have been supported by larger effect sizes in those regions for the brains which had been edited. However, such an effect was only observed for the lateral orbitofrontal cortex, and overall the differences between effect sizes for any region were evenly split between the edited and unedited methods. Therefore, it is evident that although there were differences between the two methods, editing the brain images didn't translate into our ability to detect group differences more readily with one method or the other.

## Limitations

Artifacts due to intensity inhomogeneity, head motion, reduced signal to noise ratio, and partial volume effects can all lead to reduced image quality, alterations in intensity values and, ultimately, errors in image segmentation. These issues may be magnified in higher field-strength data secondary to increases in B1 field inhomogeneity (Marques et al., 2010), potentially necessitating more manual editing of higher field-strength images. Acquiring and averaging multiple acquisitions, which improves signal-to-noise and contrast-to-noise ratios, and reduces motion artifacts, can address these issues (Kochunov et al., 2006; Winkler et al., 2010). The present analyses were based on a single sequence acquisition, which therefore constitutes a limitation to our study. Multiple sequence acquisition carries trade-offs in both scanning cost and time, which can deter researchers. In the present study, the sample consisted, in part, of school-aged children with intellectual disability and, in many cases, attention deficit hyperactivity disorder. Accordingly, we had to strike a balance between optimizing the quality of our images while maintaining a timeframe that our sample would tolerate. This may have necessitated more manual intervention to correct errors in segmentation.

Although we observed similarities in the metrics we extracted from the different regions of the brain, we did not conduct an overlap analysis to determine whether the ROIs had a high level of spatial overlap. It is possible that the regions appear to be similar numerically, but have different boundaries with one methodological approach more accurately denoting the region it represents. Another limitation is that both the 1.5T and 3T data used were manually skull stripped prior to implementing the FS pipeline: if brains were run fully automated, they would be subject to the automated skull stripping module included within FS. However, we do not believe that had a significant effect on our results, and previous research supports this notion (Fennema-Notestine et al., 2006). Our processing pipeline may have also been limited by the fact that we did not assess the quality of the images (e.g., signal to noise ratio) prior to processing the data, which may have affected the extent to which manual interventions were needed.

## Conclusions

This study is significant in that it shows that the additional time and cost necessary to manually correct the FS segmentation process does not necessarily increase one's ability to detect differences in cortical measurements between groups. Future studies should be conducted with larger and more diverse samples in order to provide additional insight into the differences between methods. In addition, since the temporal and frontal lobe contain numerous regions affected by disorders like Alzheimer disease and schizophrenia, and many of the differences we observed were within those lobes, additional research should focus on methods which can increase the segmentation accuracy specifically in those regions.

## Authors contributions

WK, IC, and CM designed the study. CM, CT, and JB completed all image processing for the study. IC and WK completed all statistical analyses of the imaging data. AR, CM, and WK wrote the manuscript. All authors revised the manuscript for accuracy and intellectual content, and all authors approved the final manuscript.

### Conflict of interest statement

The authors declare that the research was conducted in the absence of any commercial or financial relationships that could be construed as a potential conflict of interest.

## Footnotes

^1^http://web.archive.org/web/20150901150339/http://surfer.nmr.mgh.harvard.edu/fswiki.

^2^http://web.archive.org/web/20150901145928/https://surfer.nmr.mgh.harvard.edu/fswiki/FsTutorial/ControlPoints_freeview.

^3^http://web.archive.org/web/20150901150038/https://surfer.nmr.mgh.harvard.edu/fswiki/FsTutorial/WhiteMatterEdits_freeview.
