# Supplementary material for: A comparison of FreeSurfer-generated data with and without manual intervention
Source: Front Neurosci. 2015 Oct 21;9:379. doi: 10.3389/fnins.2015.00379 (PMC4612506; doi:10.3389/fnins.2015.00379)
Supplement: Supplementary file 1 [file DataSheet1.PDF]

May 2013

FreeSurfer - 1.5T

Center for Psychiatric Neuroimaging

An in depth look at the different components and methods of the Center for Psychiatric Neuroimaging's FreeSurfer project and protocol.

Upstate Medical University

Table of Contents

Overview of FreeSurfer Components....3

Step 1: PreProcessing....3

Step 2: [CROSS] FreeSurfer Images....4

Step 3: Creating [BASE] Template....5

Step 4: Creating [LONG] Scans....6

Detailed PreProcessing Protocol....7

Step 1 – Format Conversion....7

Step 2 – ACPC Alignment/Voxel Resampling....9

PreProcessing Appendix A....13

(Slicer3, AC/PC Examples, Midline Examples)....13

Appendix B - Slicer Controls....17

CPN FreeSurfer Pipeline....18

Initializing Cross-Sectional Data and Running Recon1-2....19

Running Recon3....21

Creating [BASE] Template....22

Manual Editing Interface....23

Manually Correcting Errors....25

TKMEDIT Information....26

Motion Errors....28

Pial Surface Excludes Brain Matter....29

Pial Surface Includes Non-Brain Matter....30

Hyper Intensities....31

FreeSurfer Editing Tips....32

Overview of FreeSurfer Components

Step 1: PreProcessing

Preprocessing is the first step in the FreeSurfer pipeline. The operator begins with the raw DICOM files and, using the Macintosh and Ubuntu systems, ends with a skull stripped brain aligned in standard ACPC space. In the final step the brain is re-sliced, so that the voxels are in isotropic space.

Required Files: IR_Prep image series, Skull Strip .TIFF File

Step 2: [CROSS] FreeSurfer Images

The next step involves importing the files resulting from preprocessing into FreeSurfer and creating the Cross-Sectional FreeSurfer data. FreeSurfer has different pipelines available depending on the relationship of data. If the imaging data is Cross-sectional, with all images independent from each other, then you only need to run the standard recon-all command. This Data is referred to in a variety of ways, including: the Cross Files, Cross-Sectionals, Individual Time Point data, or by the specific time point (e.g. - "Run Time 1 for Subject 28."

Required Files: **S###_rfi_iNi.nii**
Terminal Command to Create [CROSS] Data: recon-all –s S### -all
Terminal Command to Edit[CROSS] Data: recon-all –s S### -autorecon2-cp

Step 3: Creating [BASE] Template

After creating the Cross-Sectional data, the next step is merging the information from each time point into a single Base template image. The base template is a reference that is used in the next step to create the longitudinal data. The template is created with information from the cross-sectional scans, and is later used to produce the longitudinal scans.

Required Files: [CROSS] Scans for Subjects Time Points (e.g. – S014, T014)
Terminal Command to create [BASE]:
recon-all –base ### -tp S### -tp T### -tp U### -all
Terminal Command to edit [BASE]:
recon-all –base ### -tp S### -tp T### -tp U### -autorecon2-cp

Step 4: Creating [LONG] Scans

The longitudinal scans are the last step in the FreeSurfer pipeline. The longitudinal scans utilize information from the template in order to put each Subject's individual time point data into a standardized space. This provides greater accuracy when measuring different structures of the brain.

Required Files: All [CROSS] Scans for Subject, [BASE] Template for Subject
Terminal Command to create [LONG]:
recon-all –long S### ### -all

Detailed PreProcessing Protocol

Step 1 – Format Conversion

On A Computer Running Macintosh Operating System:

Mac Log In: FreeSurfer PreProcessing
Password: preprocess

1. Create FreeSurfer Folder (if subject does not have one)
    - Open PREPROCESSING folder on Desktop
    - Right Click and Select "New Folder"
    - Rename Folder with Subject ID (e.g. – 004)
    - Create Time Point Folders within Subject ID Folder (e.g. – S004, T004, U004)

2. Copy Raw Files into FreeSurfer Folder
    - In PREPROCESSING folder on Desktop open "Raw Files"
    - Select Subject "Edit" file and copy into appropriate folder in PREPROCESSING Subject Directory (e.g. – T004_Edit_CSM should be copied into 004 → T004)
    - Select Subject "IrPrep" Folder and copy into appropriate folder in PREPROCESSING subject directory (e.g. – T004_Edit_CSM should be copied into 004 → T004)

3. Open ImageJ

4. Load DICOM into ImageJ
    - File → Import → Image Sequence
    - Navigate to Subject PREPROCESSING Directory and Open IRPreP Folder within the Time Point you are working on

5. Save DICOM image in analyze (.img, .hdr) format inside of FreeSurfer folder
    - File → Save As → Analyze…
    - Navigate to time period for current subject
    - Save file in "FreeSurfer" as **S###_istack_ini**

6. Open BrainImage Java

7. Open **S###_istack_ini** (see step 4) in Brain Image Java
    - File → Open
    - Change "File Format" to "Analyze Image Volume"

- If you are just opening Brain Image Java, it may be necessary to navigate from the applications folder to the shared folder. In the load window, select the drop down menu and follow the below path.
    - Kates Lab's Power Mac G5 → Users → freesurferpreprocessing → Desktop →PREPROCESSING →### → S###
- Double click **S###_istack_ini** or highlight it and hit "open"

8. Load skull strip 1-bit Tiff
    - File → Open
    - Change "File Format" to "NIH Tiff Volume"
    - In time period folder of current subject: FreeSurfer → **S###_edit_ini**
    - Voxel Width: .9375
    - Voxel Height: .9375
    - Voxel Depth: 1.5

9. Flip Skull Strip 1-Bit Tiff
    - Select S###_edit_ini
    - Algorithms → Rotate/Flip → Flip; Hit OK.
    - Make sure "X-Axis" and "Vertical" are selected
    - Hit OK

10. Create VOI
    - Algorithms → Binary → BinaryDataCells3D to VOI
    - Hit OK

11. Load Skull Strip 1-Bit .tiff onto iStack
    - Double click S###_istack_ini
    - Select "new multiplanar viewer" and hit "OK"
    - With the Multiplanar Viewer window highlighted, Invert Color (Color → Set Inverted Colormap)
    - Double Click "VOI[#]"; Select "Multiplanar Viewer: S####_istack_ini"
    - Hit "OK"

12. Clear Non-Brain matter from istack
    - Select Multiplanar Viewer Window
    - VOI → Clear Outside to White

13. Save "S###_istack_ini"
    - Click on main Brain Image Java window and highlight "S###_istack_ini"
    - File → Save As
    - Change "File Format" to "Analyze Image Volume"
    - Save as: "**S###_rfslicer_ini**" in current subjects FreeSurfer folder.
    - Select "16-Bit"

14. After finishing every time point for a Subject, copy the entire Subject folder onto Ubuntu machine.

- Cut Subject Directory in PREPROCESSING folder on MAC (CTRL – X)
- Paste onto Jump Drive (CTRL – V)
- Bring over to Freesurfer #1
- Open /data/freesurfer/PREPROCESSING
- Cut Subject Directory in Jump Drive (CTRL – X)
- Past into /data/freesurfer/PREPROCESSING

Step 2 – ACPC Alignment/Voxel Resampling

1. Open Slicer3
   - Click yellow button in top navigation menu

2. Add "**S###_rfslicer_ini**" to Slicer3
   - File → Add Data; click Add Directory
   - Navigate to /data/freesurfer/preprocessing and select the subject folder you are working on
   - Make sure "**S###_rfslicer_ini.hdr**" is selected and the box underneath "centered" is selected as well.
   - Click Apply

3. Create ACPC Fiducial Lists
   - Under modules drop down menu highlight "All Modules" and then "ACPC Transform" and release
   - Click box next to "ACPC Line"; highlight "Create New Fiducial List" and release
   - Click box next to "ACPC Line" and highlight "Rename…" and rename fiducial list "ACPC"
   - Repeat steps above for Midline, and Output transform with the following naming convention:
     - Midline – Midline
     - Output Transform – ACPC Transform

4. Open Fiducials Module
   - Under modules dropdown menu select Fiducials and release

5. Place Anterior Commissure (AC) Fiducial (Reference Appendix A for Slicer3 Interface, Controls, and AC examples)

- Change View to "Red Slice Only Layout"
- Navigate (left and right arrow keys) until AC is visible. The AC is thick
- connecting fiber anteriorly located crossing across the right and left hemispheres.
- The AC usually appears halfway through the axial stack. Move mouse cursor over AC, and press "p" to place the fiducial.
- When choosing between two slices, select the slice with a higher "bg" value (visible on the bottom left bar of the program, **see Appendix A, Image 1**)

5. Place Posterior Commissure (PC) Fiducial (Reference Appendix A for Slicer3 Controls and PC examples)

- Select axial view and navigate (left and right arrow keys) until posterior commissure is visible. The posterior commissure appears as a thin band of connecting fibers posterior to the anterior commissure and within ~10 slices of it. Put mouse cursor in center of PC and press "p" to place fiducial.
- When choosing between two slices, select the slice with a higher "bg" value (visible on the bottom left bar of the program, **see Appendix A, Image 1**)

6. Place Midline Fiducials (Reference Appendix A for Slicer4 controls and more information on Midline)

- Change View to "Green Slice Only Layout"
- Highlight the midline list in the Fiducial module.
- Navigate (left and right arrow keys) to the most anterior or posterior section of the brain in the coronal view (Right, Green)
- Begin moving (left and right arrow keys) through the brain placing 3-4 fiducials in the interhemispheric fissure every 15-20 slicers. The interhemispheric fissure is the CSF that separates the two hemispheres of the brain. In the image, it manifests itself as a line of darker intensity voxels running roughly through the center of the brain. Alternatively it will look like "blobs," of dark intensity voxels.
- When placing fiducial points, avoid placing them in larger clumps of lower intensity voxels where it is easier to place them off of the true midline. (**See Appendix A, Image 7)**

7. Apply ACPC Transform

- In ACPC Transform module (see step 3) hit "Apply"

8. Align "**S###_rfslicer_ini**" to ACPC Space

- In modules drop down menu highlight "All Modules" then "resample scalar/vector/dwi volume" and release.
- Make sure "Input Volume" is "S###_rfslicer_ini"
- Make sure "Output Volume" is a new stack named "S###_rfslicer4_ini"

- Make sure “Transform Node” is “ACPC Transform”
- Hit “Apply.”

9. Save “S###_rfslicer4_ini”
   - File → Save
   - Select all files in save screen window
   - “Change Destination for All Selected” to subjects preprocessing folder and hit “Save Selected”

10. Open Slicer4
    - Hit Red Button on Ubuntu Toolbar

11. Load “S###_rfslicer4_ini”
    - File → Add Data
    - Choose Files(s) to Add
    - Navigate to subjects preprocessing folder and double click “**S###_rfslicer4_ini**”
    - Select “Show Options”
    - Make sure box next to “Centered” is checked
    - Hit “Ok”

12. Open Resample Scalar Volume Module
    - Spacing should be .9375, .9375, .9375
    - Interpolation Method should be “bspline”
    - Input Volume: “S###_rfslicer4_ini”
    - Output Volume: create new volume and name it “**S###_rfi_ini**”
    - Hit “Apply”

13. Save All Slicer 4 files
    - File → Save
    - Make sure each file in resulting window is selected.
    - “Change Destination for All Selected” to current subjects PREPROCESSING folder.
    - Save “**S###_rfi_ini**” as a **NiFti (.nii)** file (change file type in drop down menu located next to file name.

14. Copy RFI file into READY FOR IMPORT folder
    - Highlight “S###_rfi_ini” created in Step 14
    - Copy (CTRL – C)
    - Navigate to /data/freesurfer/PREPROCESSING/READY FOR IMPORT
    - Paste (CTRL – V)

PreProcessing Appendix A

(Slicer3, AC/PC Examples, Midline Examples)

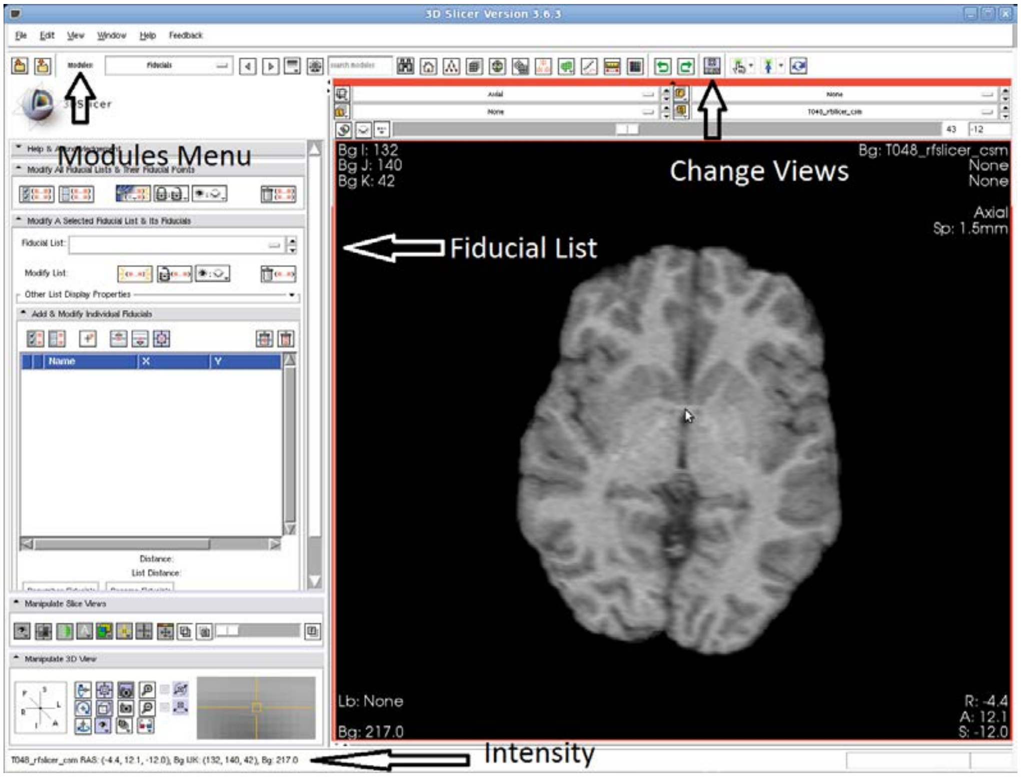

Image 1 - Slicer3 program with relevant functions labeled.

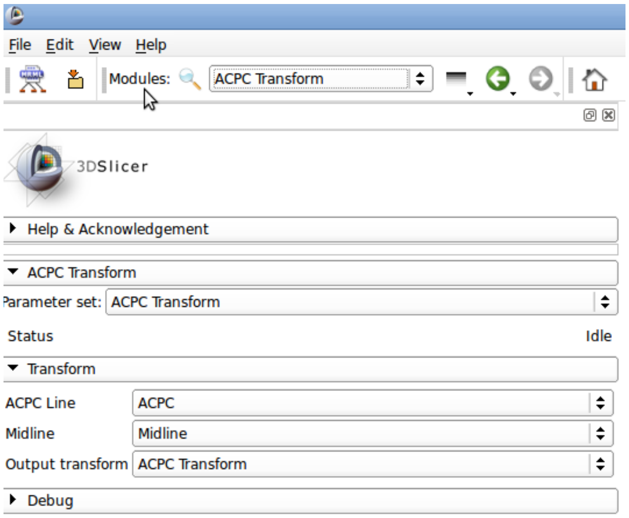

Image 2 – This is a screen shot of the ACPC Module that is used in steps 3, and 8. Above the Slicer logo (top left) you can see the modules drop down menu (currently contracted).

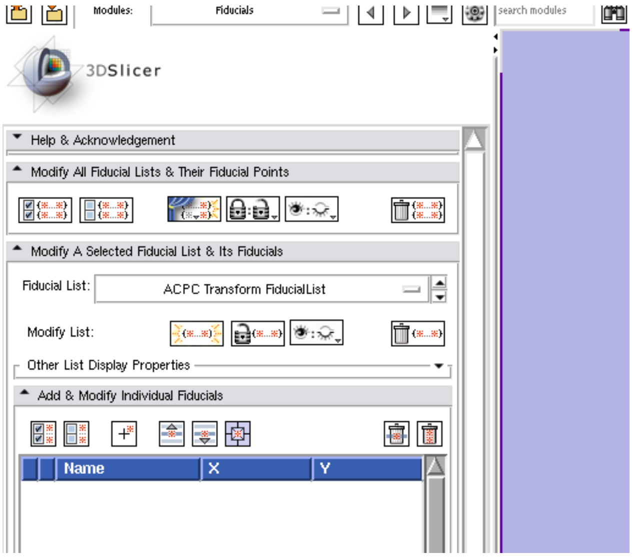

Image 3 – The fiducial module.

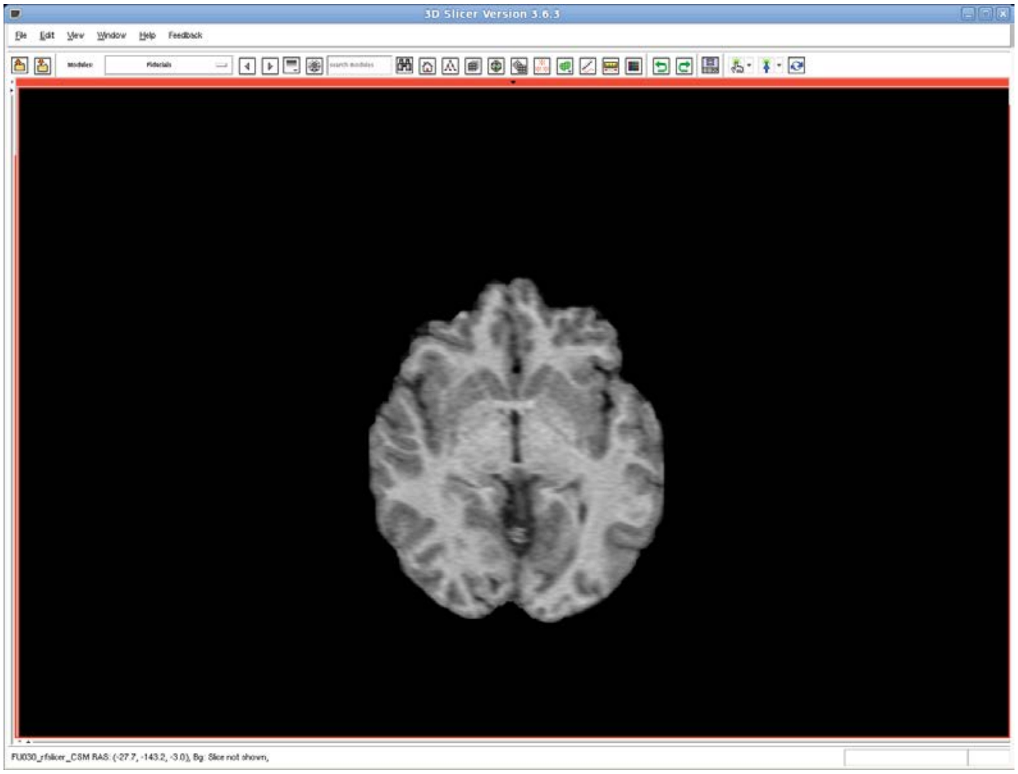

Image 4 – Above is another axial slice with both commissures present. It isn't necessary to differentiate between the two when placing your fiducials. However the anterior commissure is the thick band of fiber closer to the frontal lobe, and the posterior commissure appears more posteriorly, near the top of the cerebellum.

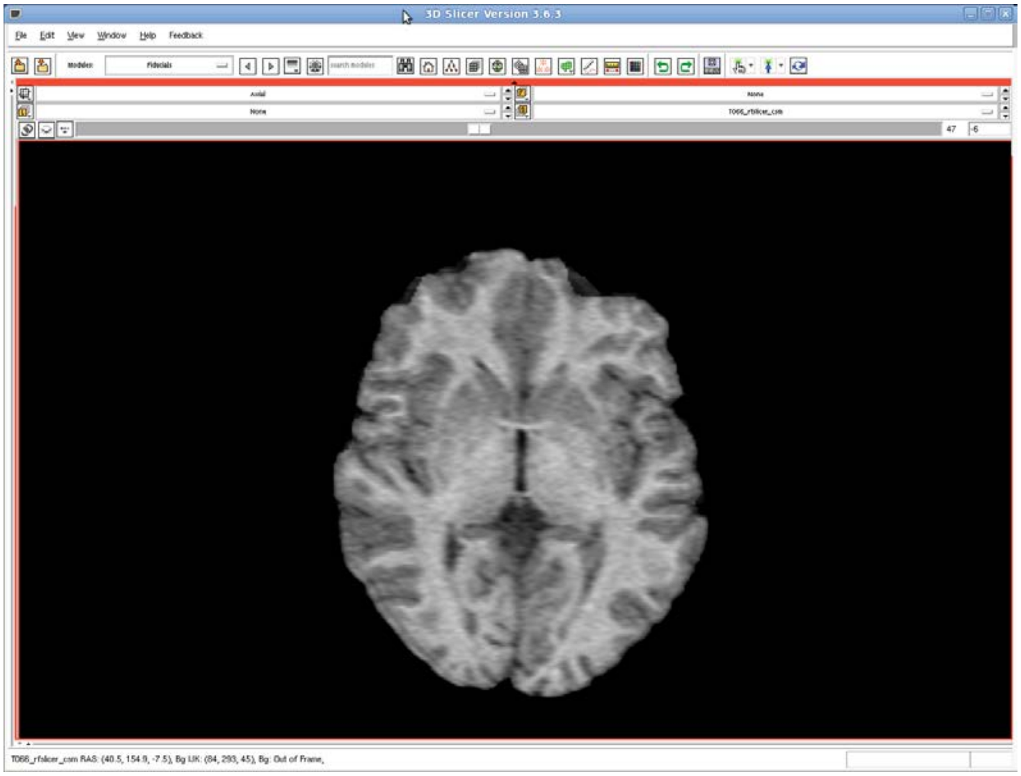

Image 5, 6 (below) – This is an axial slice showing both the anterior commissure and posterior commissure. Typically the commissures appear on different axial slices, but will look similarly to the picture above. The picture below is identical, but the commissures are labeled.

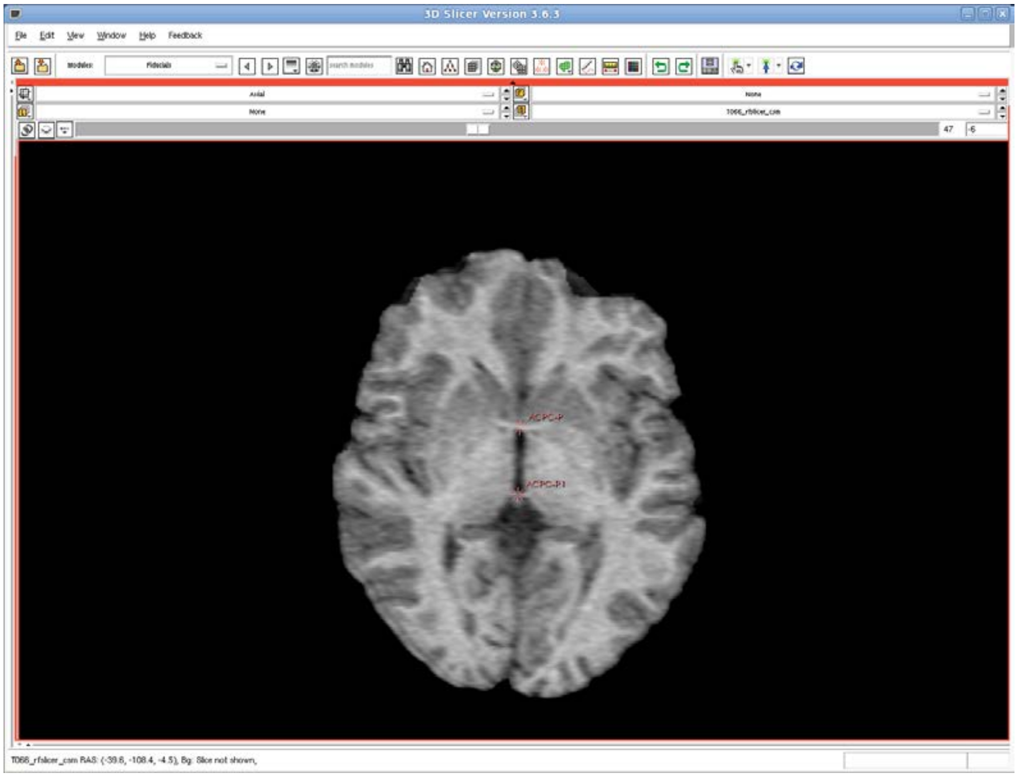

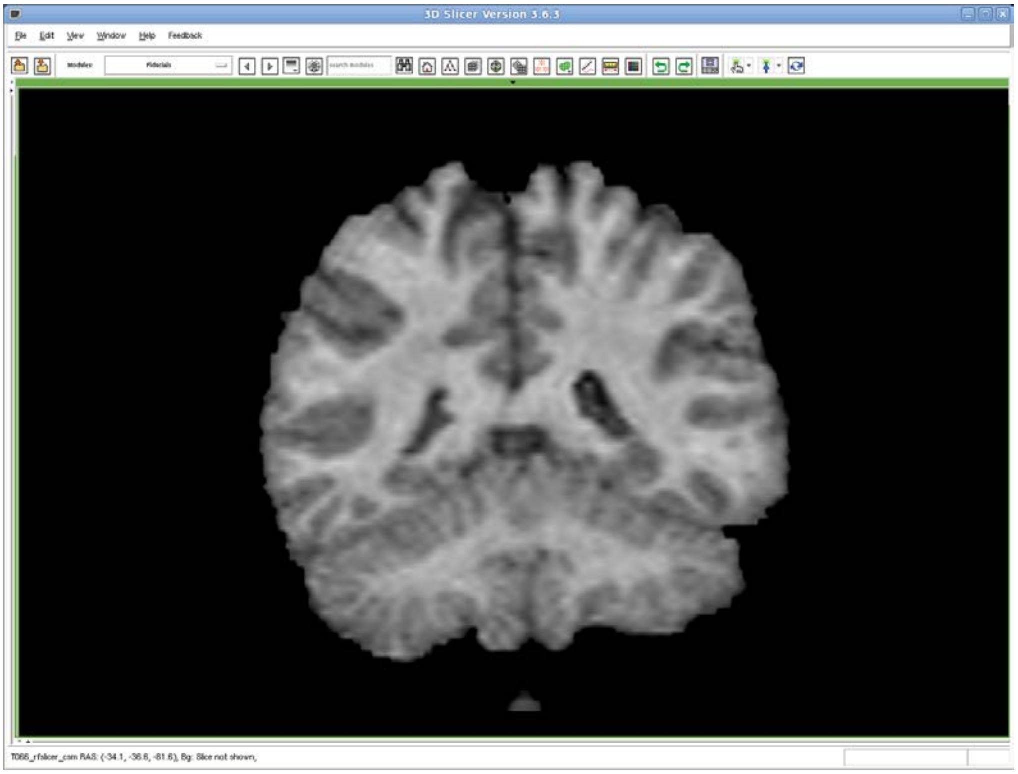

Image 7, 8 (below) – The above image shows the midline. The midline is a fissure between the two hemispheres of the brain that manifests itself as a straight line of cerebrospinal fluid (CSF) like above, or "bubbles" of (CSF) roughly aligned vertically. The image below shows fiducials placed correctly on the midline.

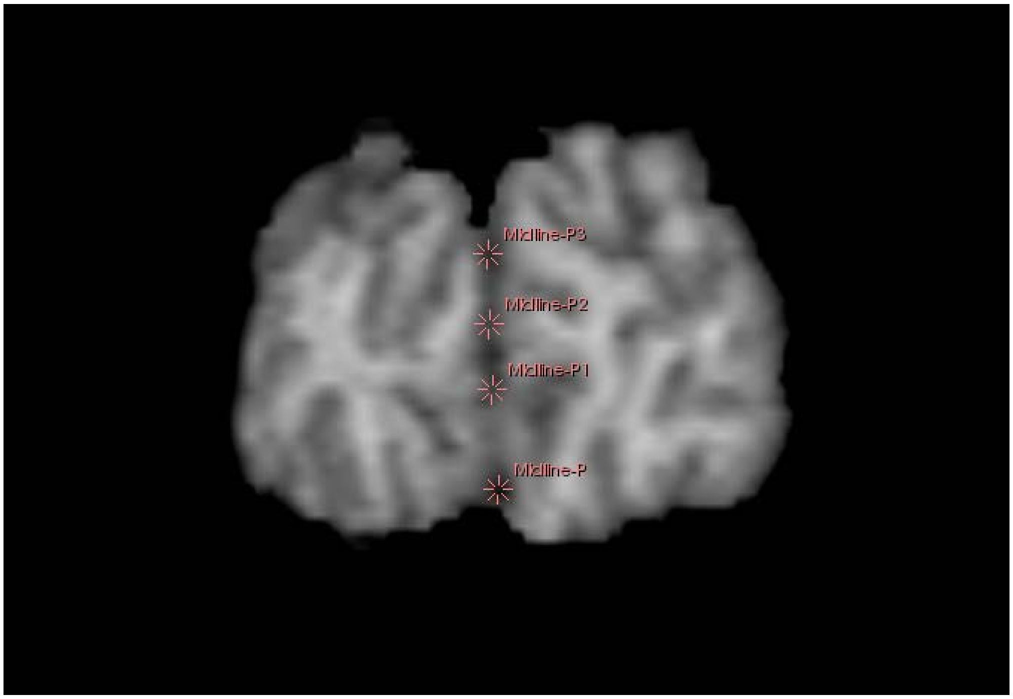

Appendix B - Slicer Controls

Arrow Keys - Navigates through the brain (left, up – anteriorly; right, down - posteriorly) when the image is selected. If you are pressing the arrow keys and not moving through the brain, click on the image with the left mouse button.

P – Places a fiducial point

Ctrl + Moving Right Mouse Button – Zooms the Image, useful if you want to get a closer look at the AC/PC or Midline

Ctrl + Moving Left Mouse Button – Drags the image around, which is useful if you've zoomed

CPN FreeSurfer Pipeline

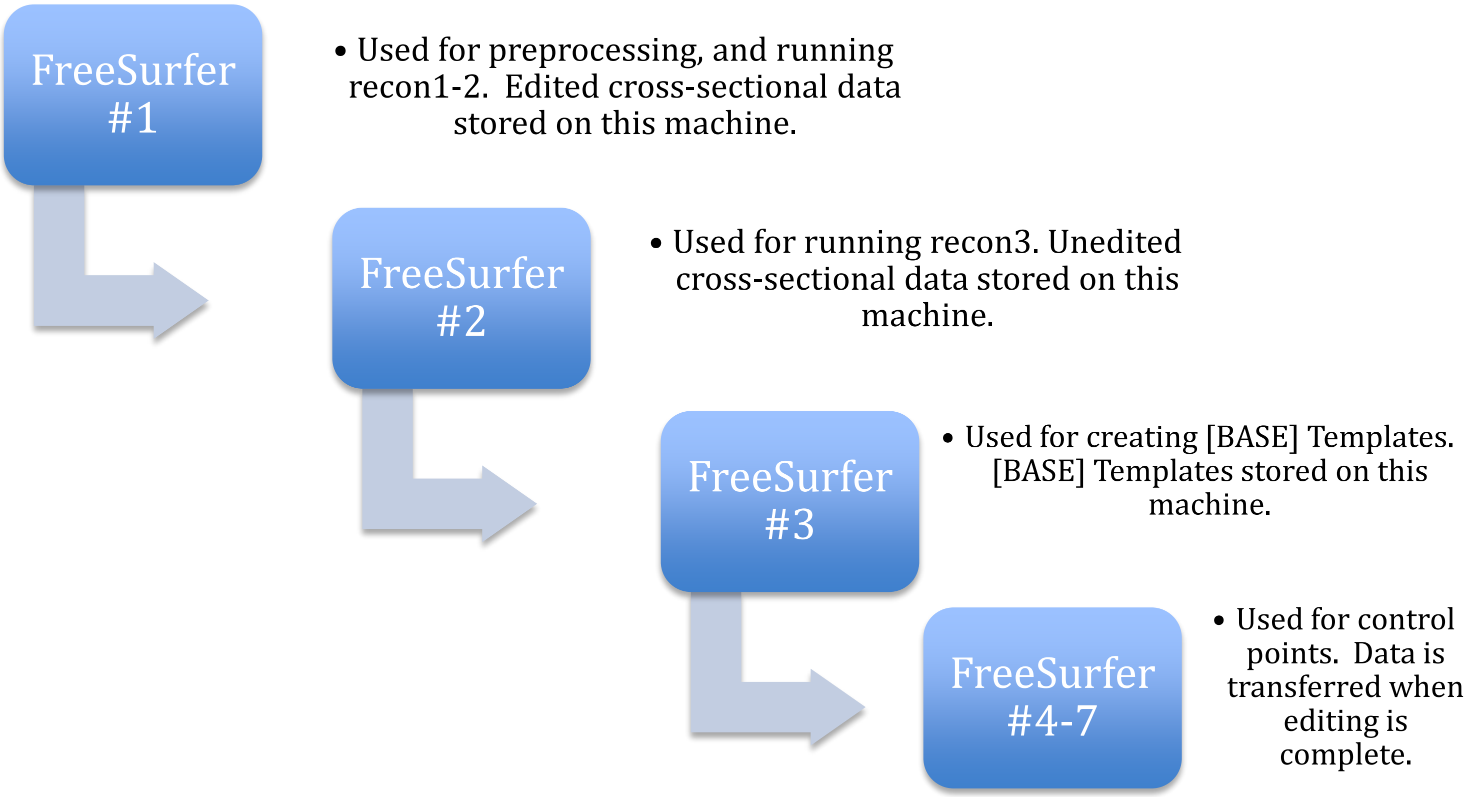

FreeSurfer Process Diagram – Shows the progression of the FreeSurfer recon-all process across multiple machines. FreeSurfer data is physically separated to ensure least amount of errors in processing. Manual Editing is done on separate machines, and returned once completed.

Initializing Cross-Sectional Data and Running Recon1-2

FreeSurfer #1

FreeSurfer #1 will be a workhorse computer, running recon1-2, and storing FreeSurfer data that will later be manually edited with control points. Completed processes on this computer are moved to FreeSurfer #2. Lab workers will be assigned a day of the week to work on FreeSurfer#1 and on that day it will be their responsibility to transfer the completed FreeSurfer data and start new recon1-2 processes.

1. If there are any subjects in the /data/freesurfer directory, Copy Completed Data to CROSSMANEDIT and FreeSurfer #2. If not skip to step 4.

- Navigate to /data/freesurfer
- Select all subject folders in directory and Copy them ([CTRL] – C or Edit → Copy)
- Open /data/freesurfer/CROSSMANEDIT and paste subject folders inside
- Open Jump drive and paste subject folders inside
- Take Jump drive to FreeSurfer #2 and paste subject folders inside /data/freesurfer/RECON3READY
- Erase Data from Jump Drive

2. Initial "Imported and Recon1-2 Started" column in "Priority Subjects" located on Google Drive.

3. Erase Subjects in /data/freesurfer

- After copying, and only after copying, move subject directories, and RFI files to the trash and erase.

4. Move Time Point "RFI" File Into Main FreeSurfer Subject Directory

- Navigate to /data/freesurfer/PREPROCESSING/S###
- Drag "S###_RFI_iNi" into "freesurfer" button located in the navigator window.

5. Initialize Subjects and Run Recon1-2

(REPEAT FOR ALL SUBJECTS PREPROCESSED AND NOT RUN THROUGH RECON1-2)

- Maximize document titled: FREESURFER1COMMAND
- Select all text ([CTRL] + A)
- Copy all text ([CTRL] + C)
- Paste into terminal
- Replace variables:
  - [RFI FILE] – Replace with Subject RFI file name. Typically in this format: **"S###_rfi_iNi.nii"**
    TIP: Hitting Tab will autocomplete a file name, so typing in your

subject number and un underscore (S###_) and then hitting tab will finish the command.
  - [SUBJECTTP] – Replace with the subject number preceded by it's time point identier (e.g. – S005, T014, U072)
- Hit Enter

Running Recon3

FreeSurfer #2

FreeSurfer #2 is also a workhorse, and is where completed Cross-Sectional data with no manual edits is stored. The Cross-sectional data is moved from FreeSurfer #2 to FreeSurfer #3, where the Base templates are created. Lab workers will be assigned a day of the way to work on FreeSurfer #2 and start and transfer FreeSurfer processes.

1. If there are any subjects in /data/freesurfer Copy Completed Data to CROSSNOEDITS and FreeSurfer #3. If not, skip to Step 4.

- Navigate to /data/freesurfer
- Select all subject folders in directory and Copy them ([CTRL] – C or Edit → Copy)
- Open /data/freesurfer/CROSSNOEDITS and paste subject folders inside
- Open Jump drive and paste subject folders inside
- Take Jump drive to FreeSurfer #3 and paste subject folders inside /data/freesurfer/READYFORBASE
- Erase data from Jump Drive

2. Erase Subjects in /data/freesurfer

- After copying, and only after copying, move subject directories to trash and erase

3. Move Ready Subjects to /data/freesurfer

- Navigate to /data/freesurfer/RECON3READY
- Select all Subject directories
- Drag Subject directories into "freesurfer" button located in the navigator window.

4. Run Recon3

(REPEAT FOR ALL SUBJECTS NOT RUN THROUGH RECON3)

- Open Terminal
- Maximize document titled: FREESURFER2COMMAND
- Select all text ([CTRL] + A)
- Copy all text ([CTRL] + C)
- Paste into terminal by clicking middle mouse button
- Replace variables:
    - [SUBJECTTP] – Replace with the subject number preceded by it's time point identier (e.g. – S005, T014, U072)

5. Initial "Recon3 Finished Copied to CROSSMANEDIT" and "Recon3 Started" column in "Priority Subjects" located on Google Drive.

Creating [BASE] Template

FreeSurfer #3

FreeSurfer #3 is the final machine in the process leading to the creation of the [BASE] templates. FreeSurfer #3 is the computer the templates are actually created, and where the unedited templates, and edited templates are stored. Templates that need to be edited will be taken from FreeSurfer #3 and brought to users individual workstations.

1. If there are any subjects in /data/freesurfer, Copy Completed Data to UNEDITEDBASE and READYFOREDITS

- Navigate to /data/freesurfer
- Select all subject folders in directory and Copy them ([CTRL] – C or Edit → Copy)
- Open /data/freesurfer/UNEDITEDBASE and paste subject folders inside
- Open /data/freesurfer/READYFOREDITS and paste subject folders inside

2. Erase Subjects in /data/freesurfer

- After copying, and only after copying, move subject directories to trash and erase

3. Move Ready Subjects to /data/freesurfer

- Navigate to /data/freesurfer/RECON3READY
- Select all Subject directories
- Drag Subject directories into "freesurfer" button located in the breadcrumbs

4. Create BASE Template

(REPEAT FOR ALL SUBJECTS NOT RUN THROUGH BASE)

- Open Terminal
- Maximize document titled: FREESURFER3COMMAND
- Select all text ([CTRL] + A)
- Copy all text ([CTRL] + C)
- Paste into terminal by clicking
- Middle mouse button
- Replace variables:
  - [SUBJECT] – Replace with the subject number (e.g. – 005, 014, 072)
  - [SUBJECTTPID] – Replace with the subject number preceded by it's time point identier (e.g. – S005, T014, U072)

Manual Editing Interface

FreeSurfer 4-7

After the BASE template has been created it is necessary to manually correct errors to produce the most accurate segmentation possible. This should be done on the computers running FreeSurfer that aren't designated FreeSurfer 1-3. When a subject is moved to a computer, the subject will remain on that computer until the editing is finished.

1. Move Data from READYFOREDITS on FreeSurfer3 to Jump Drive
   - Navigate to /data/freesurfer/READYFOREDITS
   - Select a subject in directory and Copy them ([CTRL] – C or Edit → Copy)
   - Open Jump Drive and paste subject folders inside

2. Initial Spreadsheet – Need to Elaborate, Create Spreadsheet, ONLY ONE SUBJECT AT A TIME

3. Erase Subject in READYFOREDITING
   - After copying, and only after copying, move subject directory to trash and erase

4. Move Subject to Workstation and copy to /data/freesurfer
   - Bring Jump Drive to workstation
   - Navigate to /data/freesurfer/
   - Drag folders from Jump Drive into /data/freesurfer

5. Open Subject in TKMEDIT and replace variable (see TKMEDIT information section for more information on using TKMEDIT):
   - Open Terminal and Type or Paste the following command:
     - tkmedit [subject] brainmask.mgz. lh.white –aux wm.mgz –aux-surface rh.white –tcl $FREESURFER_HOME/bin/CIBSRtkmedit.tcl
     - [SUBJECT] – Replace with the subject number (e.g. – 005, 014, 072)

6. When the brain opens, move posteriorly until there is no brain visible. Move the brain, (hit "N" and then drag) so that there is only a single hemisphere visible.

7. Go through the brain and fix all errors in the hemisphere. The section below, Manually Correcting Errors, will help you know what is an error, and how to fix it.

8. When you finish with one hemisphere, repeat the procedure with the hemisphere that hasn't been corrected.

9. When finished with both hemispheres save the control points (file --> save control points)

10. white matter mask, (file → aux mask → save aux mask)

11. brain mask (file → save main volume)

12. quit tkmedit.

13. In addition to saving the brain, if you have put down control points that may cause more errors than they fix, create a copy of the subject folder within /data/freesurfer.

- Select the subject you are trying to copy and hit CTRL – C, then CTRL – V.

11. RUN COMMAND OR TRANSFER TO OTHER COMPUTER AND RUN COMMAND????

Manually Correcting Errors

After a Subject has been run through the entire FreeSurfer pipeline, it is necessary to inspect the generated volumes and surfaces and manually correct errors. There are two volumes and two surfaces that are manually edited in FreeSurfer. The amount of manual corrections, and the type of manual corrections will vary from subject to subject.

When a subject is first loaded with the Manual Editing Interface Command (see section above), the brainmask, main, and pial surfaces are visible:

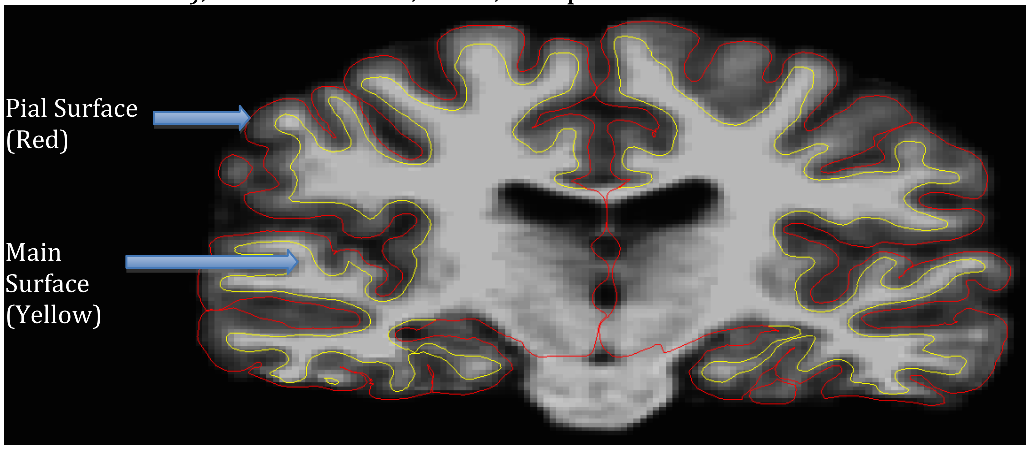

Brainmask volume with surfaces labeled.

The Pial Surface, in red, is the outermost region of the brain, and should contain all of the brain matter. The pial surface is generated from the Main Surface, which is labeled in yellow. The pial surface should contain only white matter, and is where most of the manually editing is done. In addition to the brainmask volume, the white matter mask needs to be edited as well. The white matter mask represents all of the white matter in the brain:

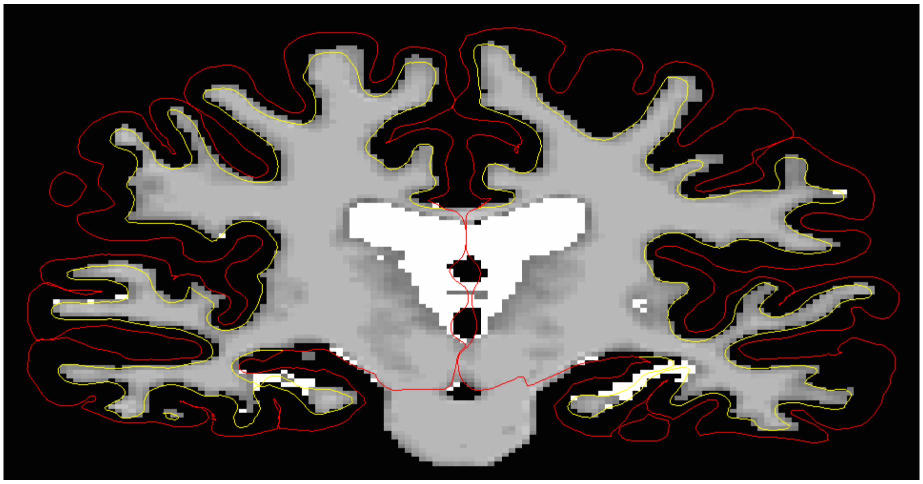

White Matter Mask

The white matter mask is often edited in conjunction with the Main Surface. Specific errors, and guidelines to correct them are provided below.

TKMEDIT Information

TKMEDIT is the name of the FreeSurfer Module used to manually correct errors. In addition to letting you erase voxels with the brush tool, TKMEDIT allows you to put down control points. A Control Point is an indicator for white matter used by the FreeSurfer algorithm. A control point should never be placed on a voxel with an intensity value less than 100, or more than 110. This section contains information on how to interact with TKMEDIT and use it to correct manual errors found in FreeSurfer data.

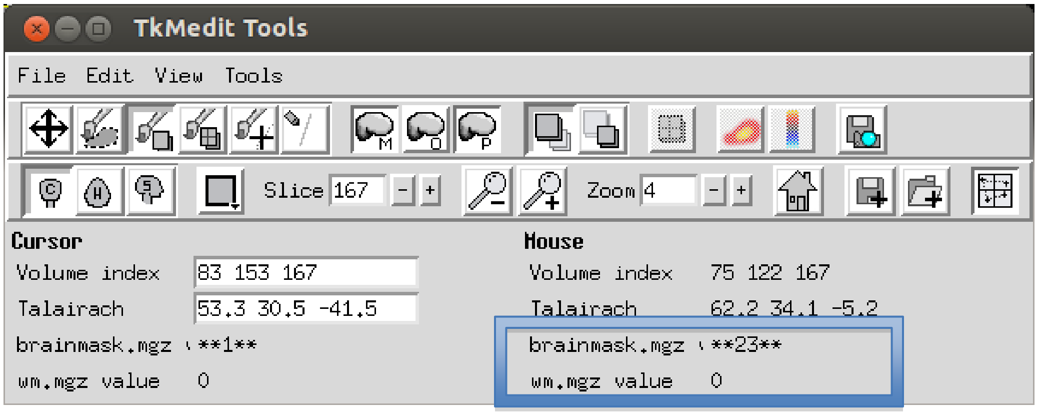

1. Use the TKMEDIT Toolbar to determine intensity values when placing control points.

2. Use the Brush Info toolbox to change editing parameters for volumes:

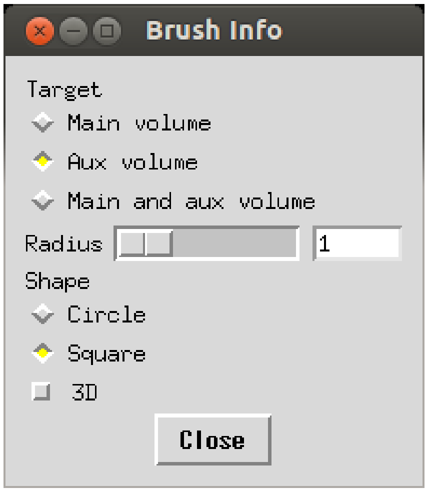

Brush Info Box: You can select which volume to edit with the brush info box, and the size and shape of your brush. Main Volume is the brainmask.mgz, and typically Aux volume is the white matter mask.

3. Use Hot Keys to navigate through the brain and manually correct errors.

- Move through the brain - [←], [→]
- Switch Brain Volume
  - [CTRL] + [1] – Switch to brainmask.mgz
  - [CTRL] + [2] – Switch to wm.mgz
- Edit Voxels (for volume editing) – [A]
  - [Right Mouse Button] – Erase
  - [Center Mouse Button] – Add white voxels
- Add/Remove Control Points – [T]
  - [Right Mouse Button] – Remove Control Point
  - [Center Mouse Button] – Add Control Point
- Place Location Indicator
  - [Left Mouse Button] – Places a purple target on a voxel so that it can be viewed in different views
- Switch Views
  - X – Switch to Sagittal View
  - Y – Switch to Axial View
  - Z – Switch to Coronal View
- Move Brain Slice (To rearrange within window)
  - [N] and Click and Drag on Brain

Motion Errors

If a subject moves during their scan, motion artifacts are the result. These artifacts, which appear as groups of higher intensity voxels around the lateral regions of the brain, draw the Main Surface out. To correct for this type of error, erase the high intensity pixels in the white matter mask, and place control points on nearby white matter. When placing control points, they should also be on voxels where the brainmask.mgz intensity value is over 100, and make sure there is adequate distance, and contrast between the voxels you are placing the control points on and the motion artifacts.

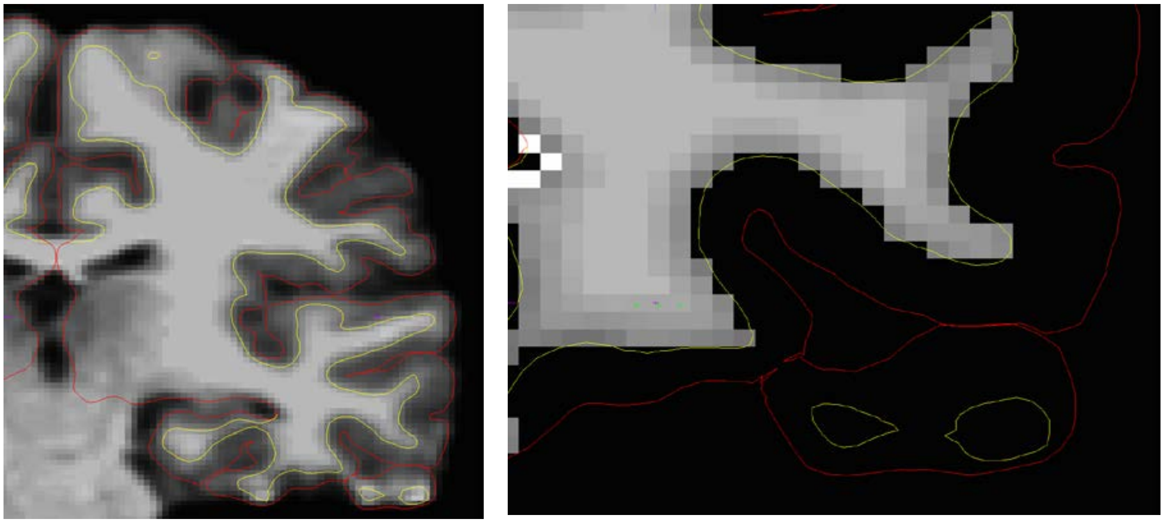

Example 1 – In the image to the left, you can see the main surface including a motion artifact in the lateral area of the temporal lobe. The image on the left, of the edited white matter mask, shows that white matter has been removed, and control points have been placed.

Pial Surface Excludes Brain Matter

The pial surface should contain all brain matter, and follow the outside of the brain accurately. Occasionally the Pial Surfaces incorrectly excludes brain matter, typically around the superior temporal gyrus of the temporal lobe, and needs to be manually corrected. To fix this type of error, place control points on white matter as closely adjacent to the excluded brain matter as possible. When placing control points, they should also be on voxels where the brainmask.mgz intensity value is over 100, and make sure there is adequate distance, and contrast between the voxels you are placing the control points on and the motion artifacts.

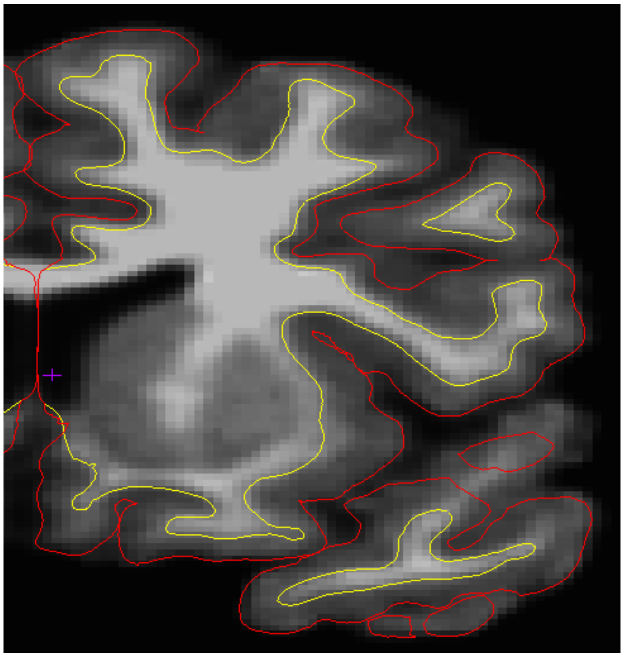

Example 1 – If the pial surface extends too far, and includes non-brain matter edit the brain.msk. To do this, in the brush info window, change the target from aux volume, to main volume and using the edit voxels function ("A") remove any non-brain matter that's been erroneously included.

Pial Surface Includes Non-Brain Matter

In the more anterior slices of the brain, occasionally the Pial Surface will include voxels that are not brain matter. These voxels are typically lower intensity than the rest of the included voxels, and should be erased.

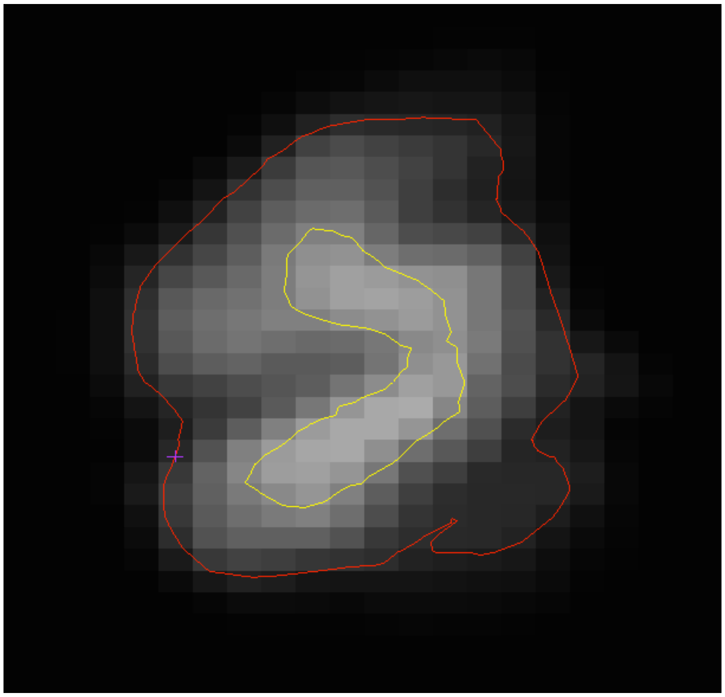

Example 1 - In the above example, the pial surface includes sagittal sinus, giving an inaccurate representation of the whole brain volume. The error, which can be seen in the medial inferior portion of the brain, should be erased so it looks like the brain below.

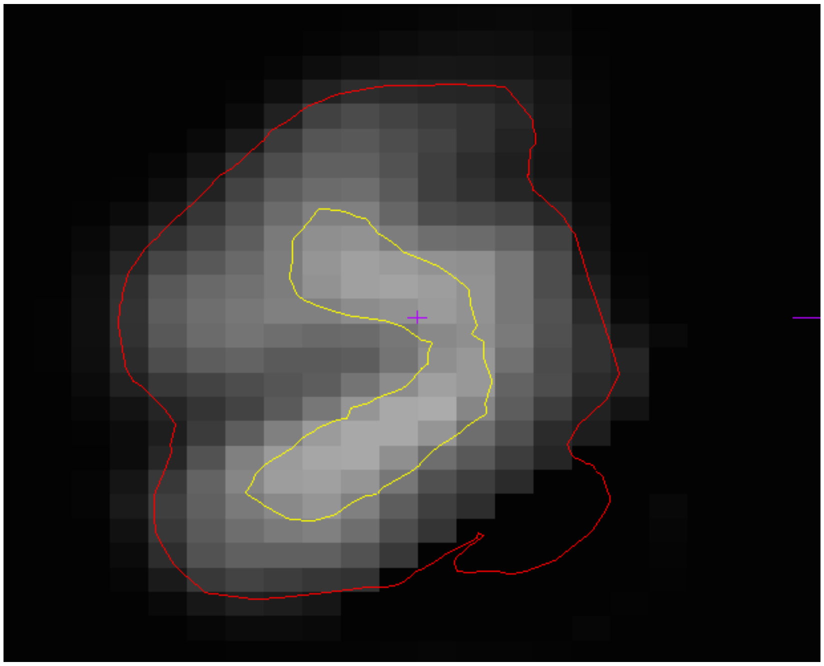

Hyper Intensities

A clumping of high intensity voxels will affect

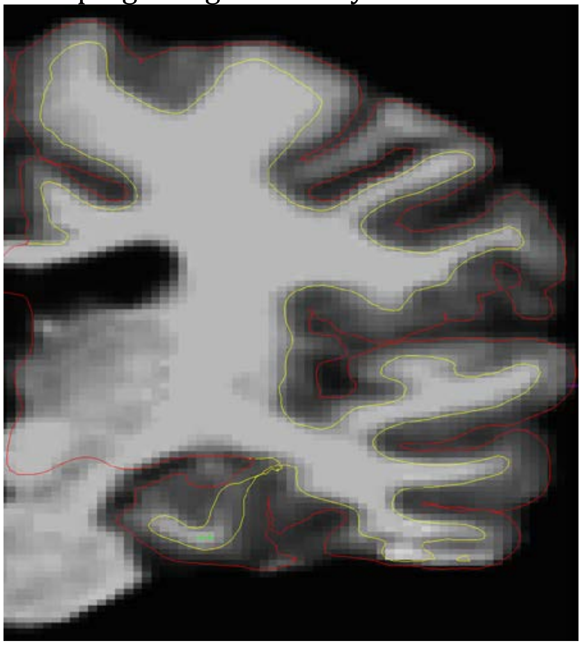

When you encounter an error, place a few control points before the error, some during (if you can find a good place to put them), but the majority of the control points should go on slices that look exactly as you want your error to look once it is corrected.

FreeSurfer Editing Tips

Scan along the outside of the brain; this is where most of the errors occur.

Look for contrast when preparing to add control points; you want large contrast between white and gray matter.

If a voxel has an intensity greater than 110, do not place a control point (it will only bring the intensity down).

Look for voxels with intensities as close to 110 as possible when looking for areas to place control points.

Control points are not always placed right on the problem you are trying to correct.

When adding white matter, first add matter where it is missing by using the middle mouse button. Then add an ample amount of control points on adjacent surfaces.

The coronal view should be the default view. If you are having trouble confirming if something is motion or not, switch to the horizontal axial or sagittal view.

If you add control points that you are unsure of or if you add a lot of control points, make a copy of the subject folder so that if your attempts at editing fail, the damage is not permanent.

When questioning whether or not something is motion, look at the slice before and after. If the motion in question falls within an area where there was white matter on the slice before/after, then it's likely not motion.

If white matter is seen on the last few slices of the brain, it's probably an error. Switch to either the sagittal or horizontal axial view and place control points in one of these views.

If you're trying to bring a surface out, place control points farther away; when bringing a surface in, control points should be more in land.
