# Supplementary material for: A comparison of FreeSurfer-generated data with and without manual intervention
Source: Front Neurosci. 2015 Oct 21;9:379. doi: 10.3389/fnins.2015.00379 (PMC4612506; doi:10.3389/fnins.2015.00379)
Supplement: Supplementary file 2 [file DataSheet2.PDF]

June 2013

FreeSurfer – 3.0T

Center for Psychiatric Neuroimaging

Upstate Medical University

Table of Contents

TKMEDIT Information 3
Downloading Data and Segmentation/Artifact Inspection 5
Manual Editing Interface 8

Manually Correcting Errors 10

Hyper Intensities 12
Main Surface Includes Gray Matter 14
White Matter Excluded from Main Surface 15
Pial Surface Excludes Gray Matter 16
Pial Surface Includes Non-Gray Matter 17
Bridge 19
Motion 20
Frontal and Temporal Lobe Specific Editing 21

TKMEDIT Information

TKMEDIT is the name of the FreeSurfer Module used to manually correct errors. In addition to letting you erase voxels with the brush tool, TKMEDIT allows you to put down control points. A Control Point is an indicator for white matter used by the FreeSurfer algorithm. A control point should never be placed on a voxel with an intensity value less than 100, or more than 110. This section contains information on how to interact with TKMEDIT and use it to correct manual errors found in FreeSurfer data.

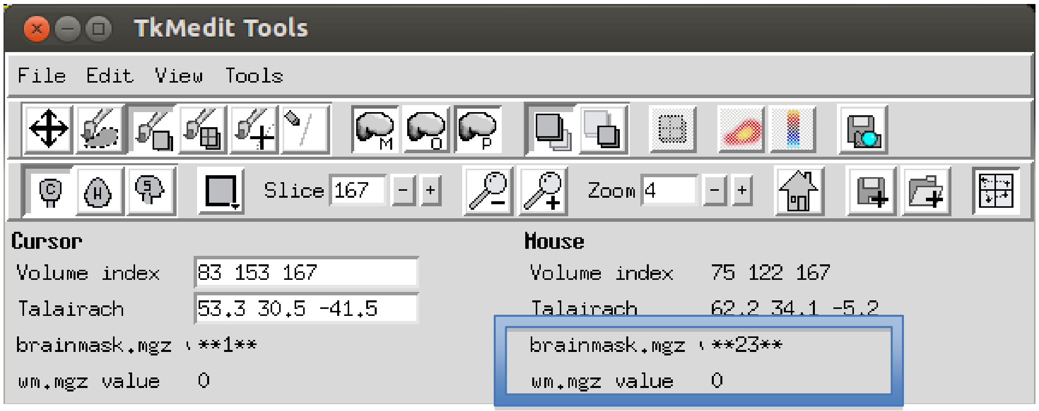

1. Open TKMEDIT by highlighting the command in the Manual Editing Interface file located in the /data/freesurfer directory of your computer.

2. Use the TKMEDIT Toolbar to determine intensity values when placing control points.

3. Use the Brush Info toolbox to change editing parameters for volumes:

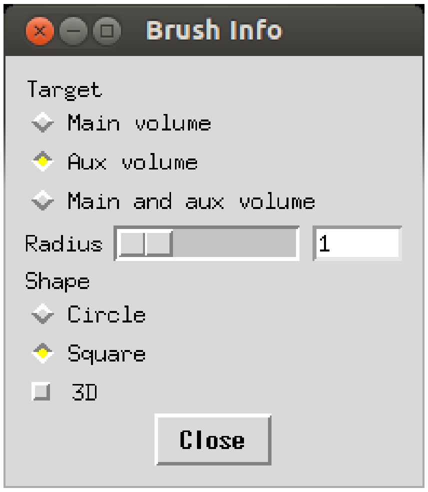

Brush Info Box: You can select which volume to edit with the brush info box, and the size and shape of your brush. Main Volume is the brainmask.mgz, and typically Aux volume is the white matter mask.

4. Use Hot Keys to navigate through the brain and manually correct errors.

- Move through the brain - [←], [→]
- Switch Brain Volume
  - [CTRL] + [1] – Switch to brainmask.mgz
  - [CTRL] + [2] – Switch to wm.mgz
- Edit Voxels (for volume editing) – [A]
  - [Shift] + [Right Mouse Button] – Erase groupings of similar intensity voxels
  - [Right Mouse Button] – Erase
  - [Center Mouse Button] – Add white voxels
- Add/Remove Control Points – [T]
  - [Right Mouse Button] – Remove Control Point
  - [Center Mouse Button] – Add Control Point
- Place Location Indicator
  - [Left Mouse Button] – Places a purple target on a voxel so that it can be viewed in different views
- Switch Views
  - X – Switch to Sagittal View
  - Y – Switch to Axial View
  - Z – Switch to Coronal View
- Move Brain Slice (To rearrange within window)
  - [N] and Click and Drag on Brain

Downloading Data and Segmentation/Artifact Inspection

1. Check FreeSurfer Time 4 Spreadsheet to find out which subjects still need to be edited. Subjects that still need manual editing will have no initials in the "Downloaded to FreeSurfer Computer #" column.

- Go to drive.google.com
- Log into Lab Google Account:
  - Username: FreeSurferCPN
  - Password: fr33surfer
- Open **FreeSurfer Time 4 Spreadsheet**

2. Copy Subjects onto the jump drive from the windows partition of a network computer at the address: //fs2/FS2DATA3/KATES3/FREESURFER_TIME4/Freesurfer5.1/RECON1_RECON2_NOEDITS

3. Move Subject to Workstation and copy to /data/freesurfer, initial "Downloaded to FreeSurfer Computer #" in the FreeSurfer Time 4 spreadsheet.

- Bring Jump Drive to workstation
- Navigate to /data/freesurfer/
- Drag folders from Jump Drive into /data/freesurfer

4. Open Document in /data/freesurfer titled "Segmentation and Artifact Inspection" and paste contents into a terminal window, replacing [SUBJECT] with the subject you are currently working on.

5. After hitting [ENTER], maximize the TKMEDIT window that opens, the brain should be colored:

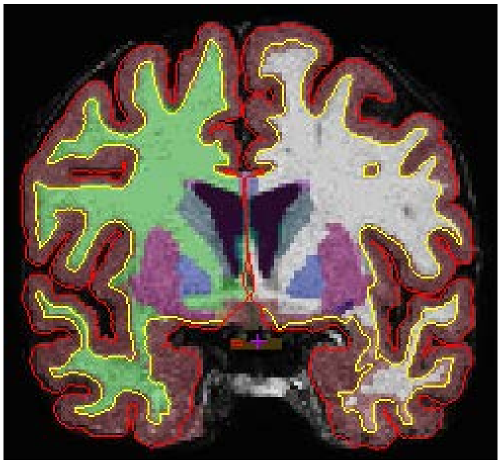

6. Scroll {[<-], [->]} through the brain until no brain matter is present. Scroll in the reverse direction looking for problems with the reconstruction process, surfaces, and segmentation that would render the brain unusable.

7. Talaraich Error – Caused by poor registration during the talaraich step in recon1. The brain will appear off kilter, like the picture below (in axial view), if there is a talaraich error, than the brain should be re-run through recon1-2:

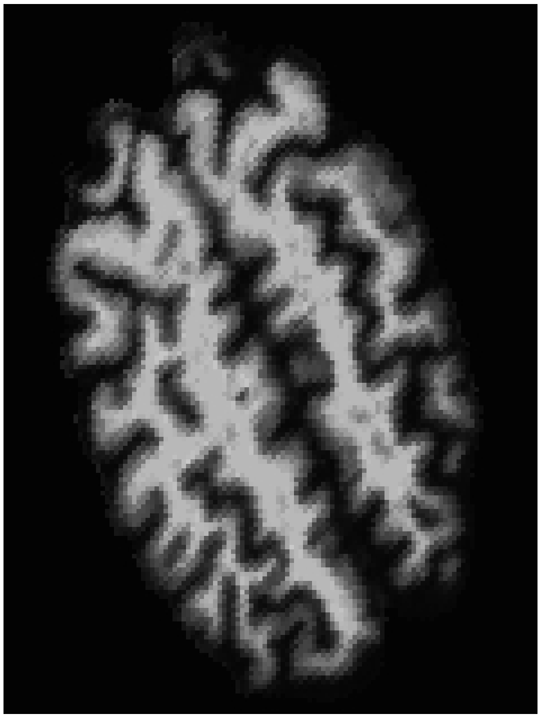

8. Segmentation Errors – Segmentation errors are caused by poor skull stripping. Regions with a segmentation error will have colored label

maps that are outside of the brain. Make a note of the coronal slice (last number after "Volume Index" in TKMEDIT toolbar) in the "Segmentation and Artifact Inspection" column of the FreeSurfer Time 4 Spreadsheet. Below are examples of segmentation errors:

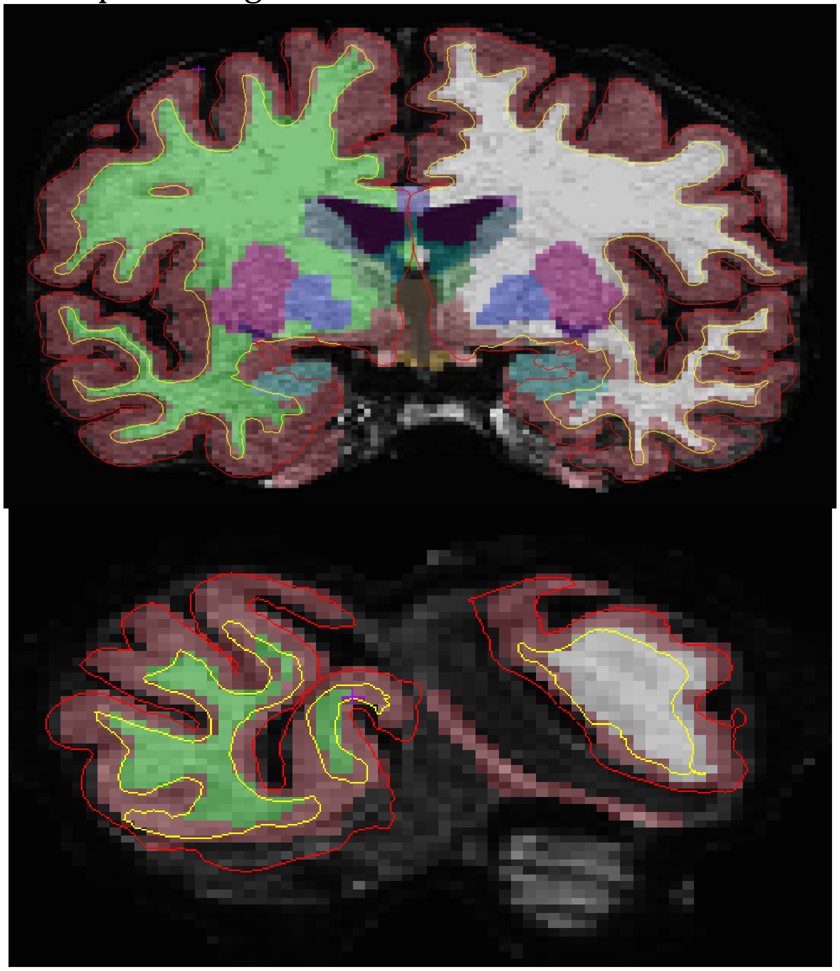

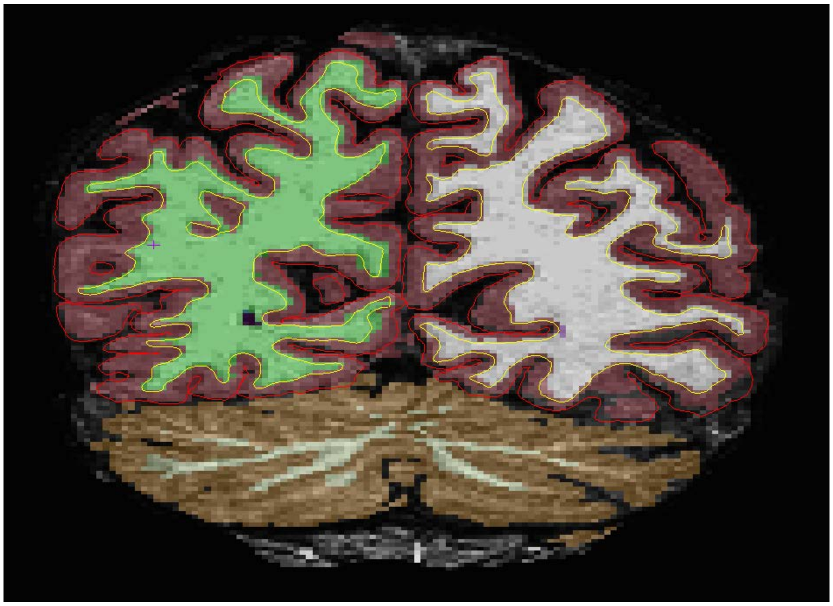

Segmentation Errors should be erased using TKMEDIT volume editing tool [A] and the brush info box (see TKMEDIT info).

Manual Editing Interface

1. Open Manual Editing Interface Document in FreeSurfer subject directory and paste command into new terminal window. Replace [Subject] with subject to be edited.

2. When the brain opens, move posteriorly until there is no brain visible. Move the brain, (hit "N" and then drag) so that there is only a single hemisphere visible.

3. Go through the brain and fix all errors in the hemisphere. The section below, Manually Correcting Errors, will help you know what is an error, and how to fix it.

4. When you finish with one hemisphere, repeat the procedure for the hemisphere that hasn't been corrected.

5. When finished with both hemispheres save the control points (file --> save control points)

6. Save the changes made to the two masks

- White matter mask - file → aux mask → save aux mask
- Brain mask - file → save main volume)
- If the option to save is grayed out, than the volume was not edited.

7. Quit tkmedit.

8. In addition to saving the brain, if you have put down control points that may cause more errors than they fix, create a copy of the subject folder within /data/freesurfer. If you aren't sure about the effect that a control point is going to have, you should make a copy of the subject folder.

- Select the subject you are trying to copy and hit CTRL – C, then CTRL – V.

9. Open "Time 4 Editing Run" document if you corrected errors and need to see the results. Highlight the text in the document and paste into a new terminal window. Replace [Subject] with the current subject being edited. Hit [Enter] to start process.

10. If all editing has been completed, open "Time 4 Final Run" document. Highlight the text in the document and paste into a new terminal window. Replace [Subject] with the current subject being edited. Hit [Enter] to start process.

Manually Correcting Errors

After a Subject has been run through the entire FreeSurfer pipeline, it is necessary to inspect the generated volumes and surfaces and manually correct errors. There are two volumes and two surfaces that are manually edited in FreeSurfer. The amount of manual corrections, and the type of manual corrections will vary from subject to subject.

When a subject is first loaded with the Manual Editing Interface Command (see section above), the brainmask, main, and pial surfaces are visible:

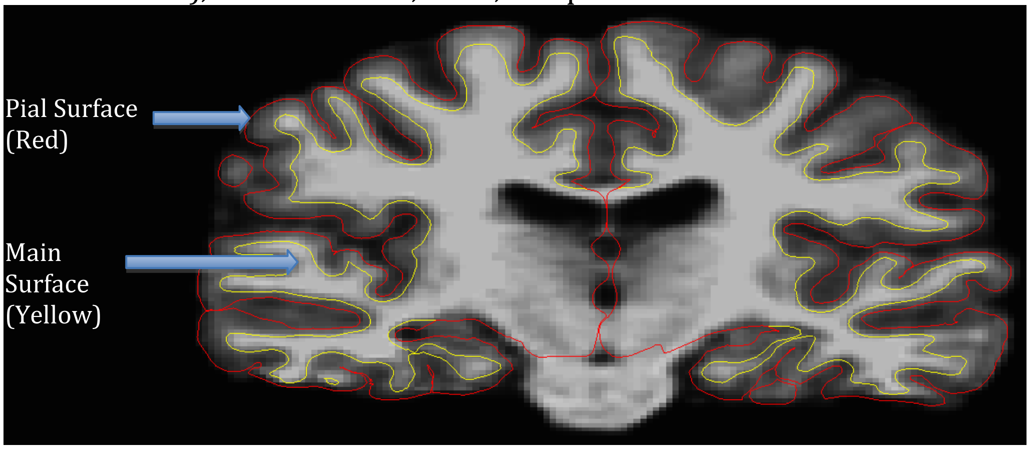

Brainmask volume with surfaces labeled.

The Pial Surface, in red, is the outermost region of the brain, and should contain all of the brain matter. The pial surface is generated from the Main Surface, which is labeled in yellow. The pial surface should contain only white matter, and is where most of the manually editing is done. In addition to the brainmask volume, the white matter mask needs to be edited as well. The white matter mask represents all of the white matter in the brain:

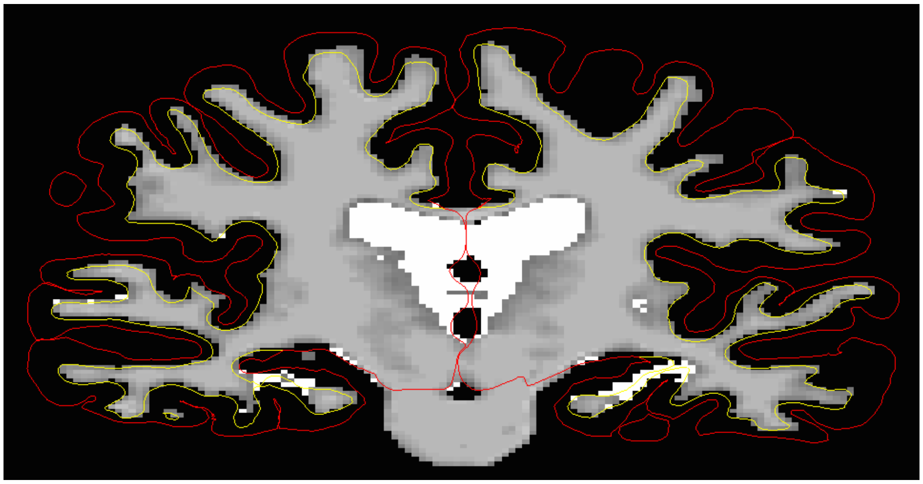

White Matter Mask

The white matter mask is often edited in conjunction with the Main Surface. Specific errors, and guidelines to correct them are provided below.

Hyper Intensities

A clumping of high intensity voxels can draw the white matter surface beyond the boundary it's supposed to follow. These high intensity voxels are typically found in the temporal and orbitofrontal regions of the brain. When the hyper intensity is distinct and separate from the white matter surface, it should be erased in the white matter mask. Control points should be placed on the adjacent white matter tract on slices where the hyper intensity is not present.

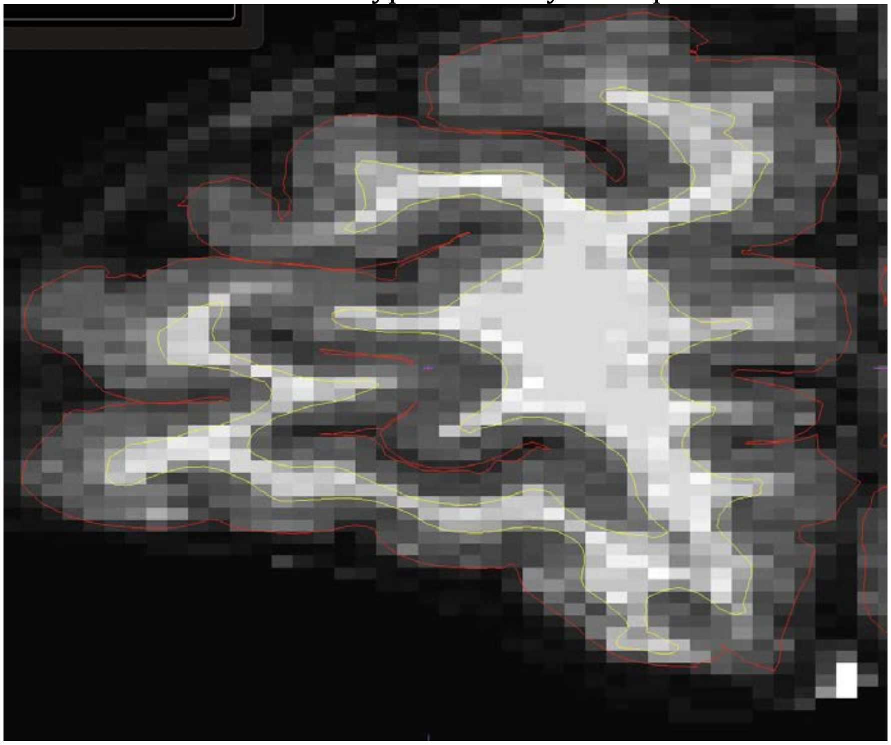

Example 1 – A hyper intensity in the orbitofrontal area is causing the main surface to incorrectly delineate the white matter. To fix this error, the underlying white matter mask should be erased, and control points should be put down on nearby slices that are correct. Example slices are below.

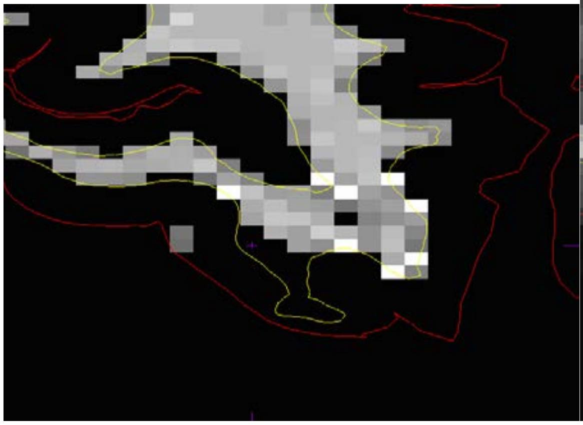

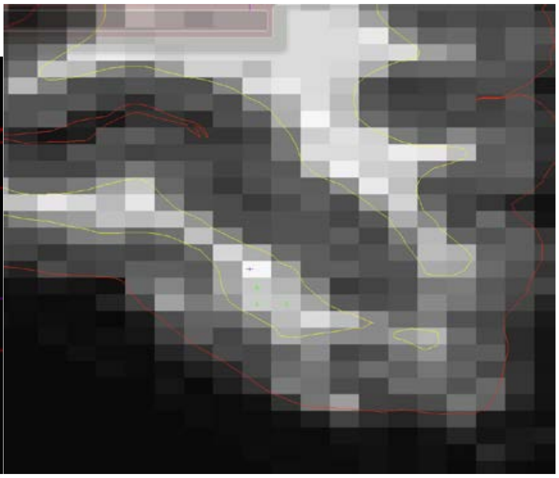

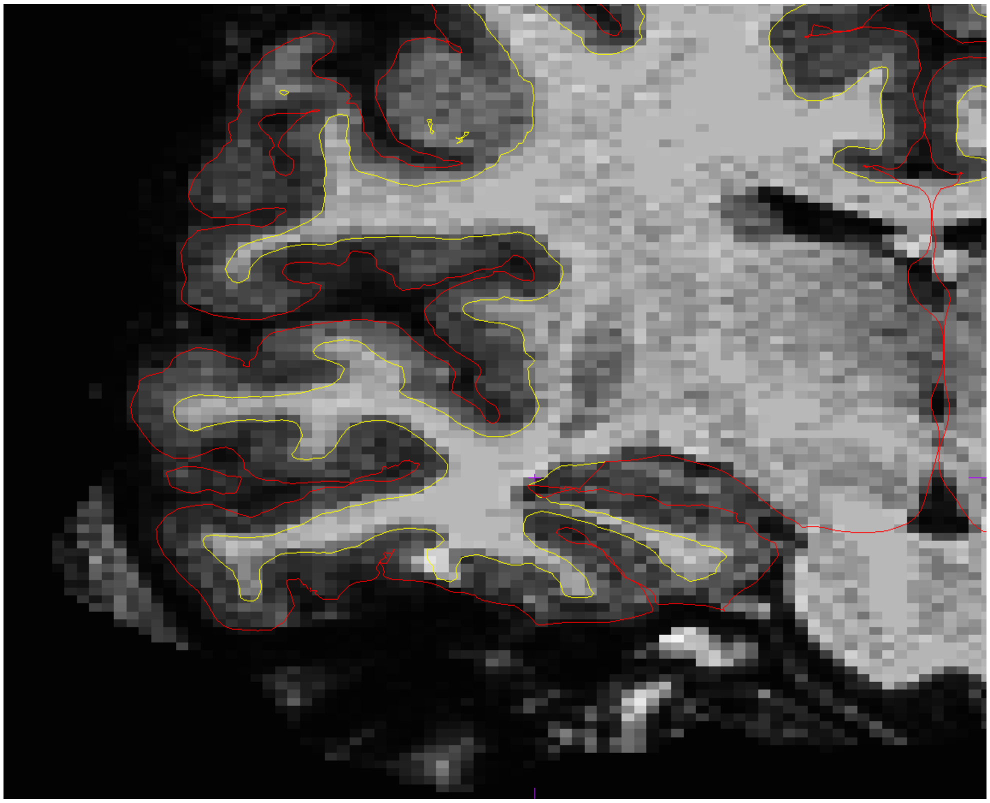

Example 2 – In Example 2, a more subtle hyper intensity error is present in the inferior temporal lobe.

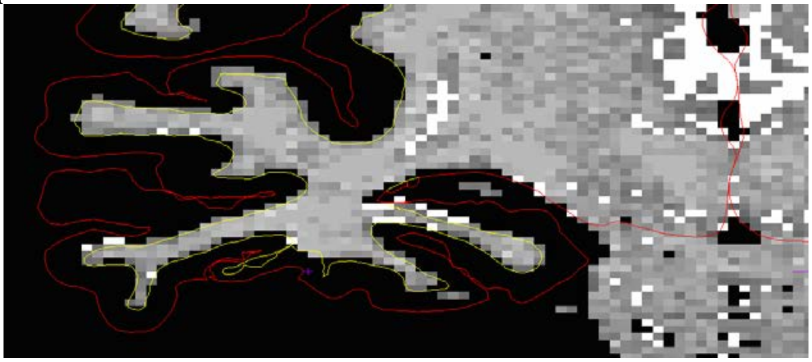

Fix 1 – Erase the pixels underlying the hyper intensity, on every slice where present.

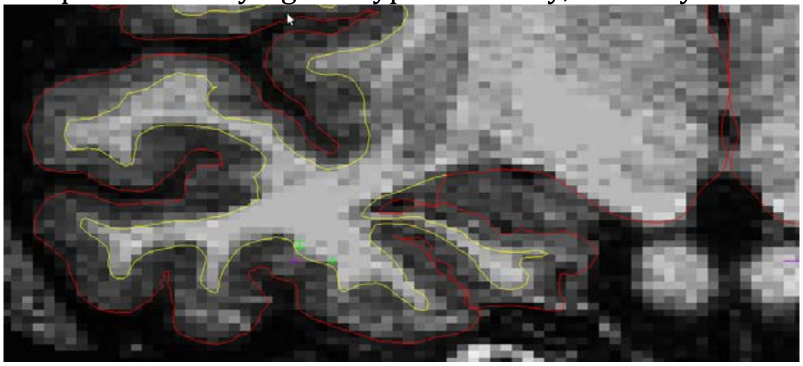

Fix 2 – Place control points on white matter near the hyper intensity, on slices where the hyper intensity is not present.

Main Surface Includes Gray Matter

Occasionally the White Matter Surface (Yellow) will include gray matter voxels, which are typically excluded. To correct this error, edit the white matter mask (CTRL + 2) and remove all white matter underlying an error. In addition, place control points around the error on adjacent white matter tracts.

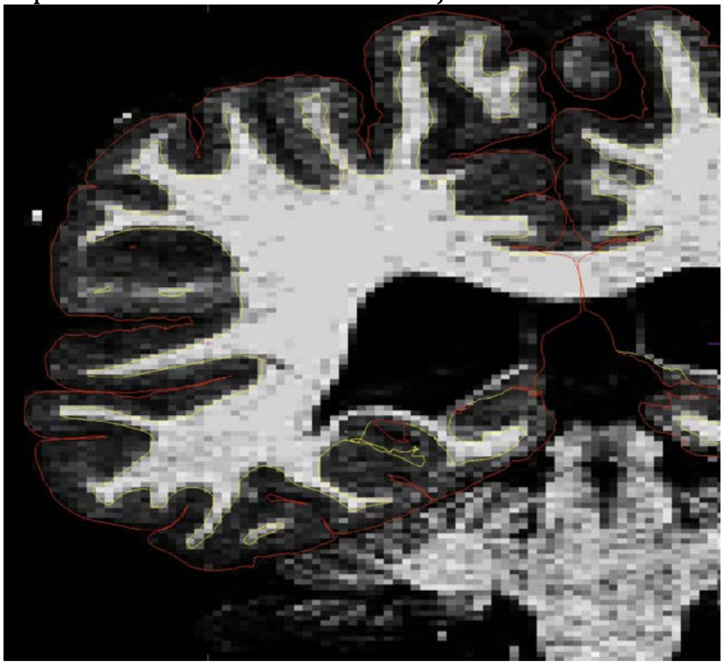

Example 1 – The white matter surface is incorrectly including gray matter near the Parahippocampal Gyrus. To fix this error, the white matter mask should be edited so there are no voxels underlying the error. Control points should be placed on the adjacent white matter tracts in appropriate locations, as seen below.

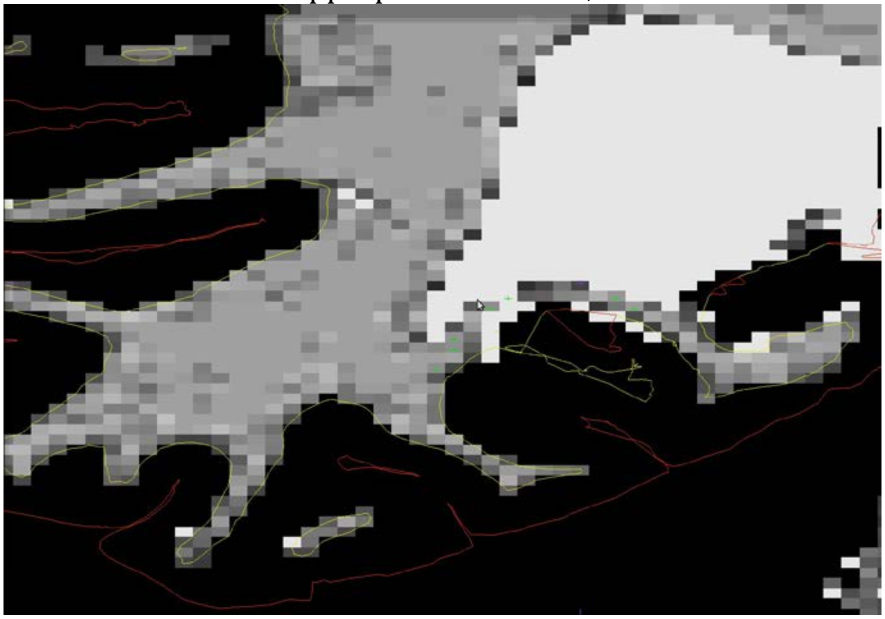

White Matter Excluded from Main Surface

When the main surface (yellow) fails to include white matter that should be included the affected structures measurements will be inaccurate. This type of error usually occurs in the anterior sections of the temporal lobe. Adding pixels to the white matter mask, and placing control points on the brain mask on the white matter voxels that have been excluded will include the white matter after recon2 has been run again.

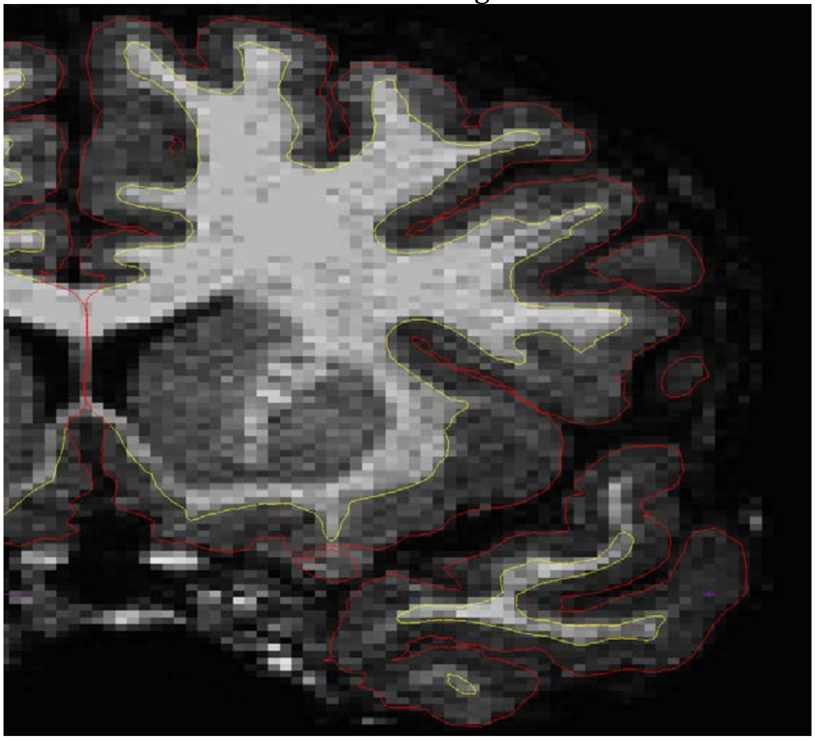

Example 1 – In the lateral section of the temporal lobe, white matter voxels are clearly excluded from the main surface. To fix the error, add control points on the slice where the white matter is included, and add white matter pixels to the white matter mask. On slices adjacent to the error, also add white matter pixels and place control points, shown in the picture below.

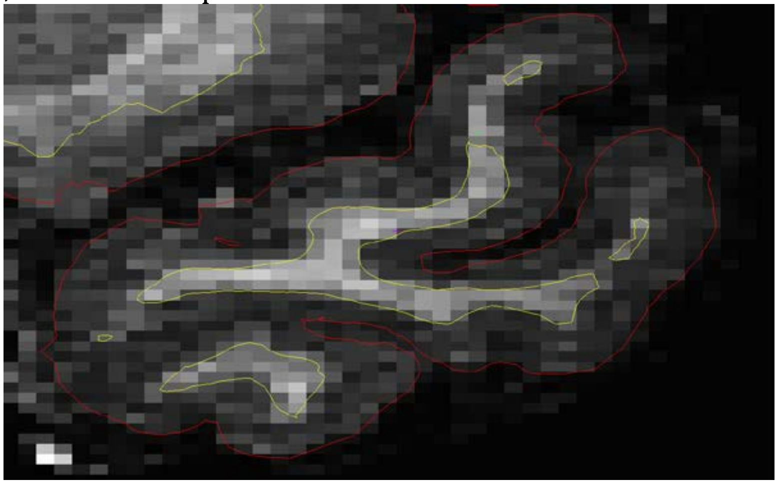

Pial Surface Excludes Gray Matter

A common error in the anterior sections of the temporal lobe and orbitofrontal lobes is for the pial (red) surface to exclude gray matter. To fix this type of error, control points should be placed on nearby white matter tracts to push the pial surface to include the missing gray matter.

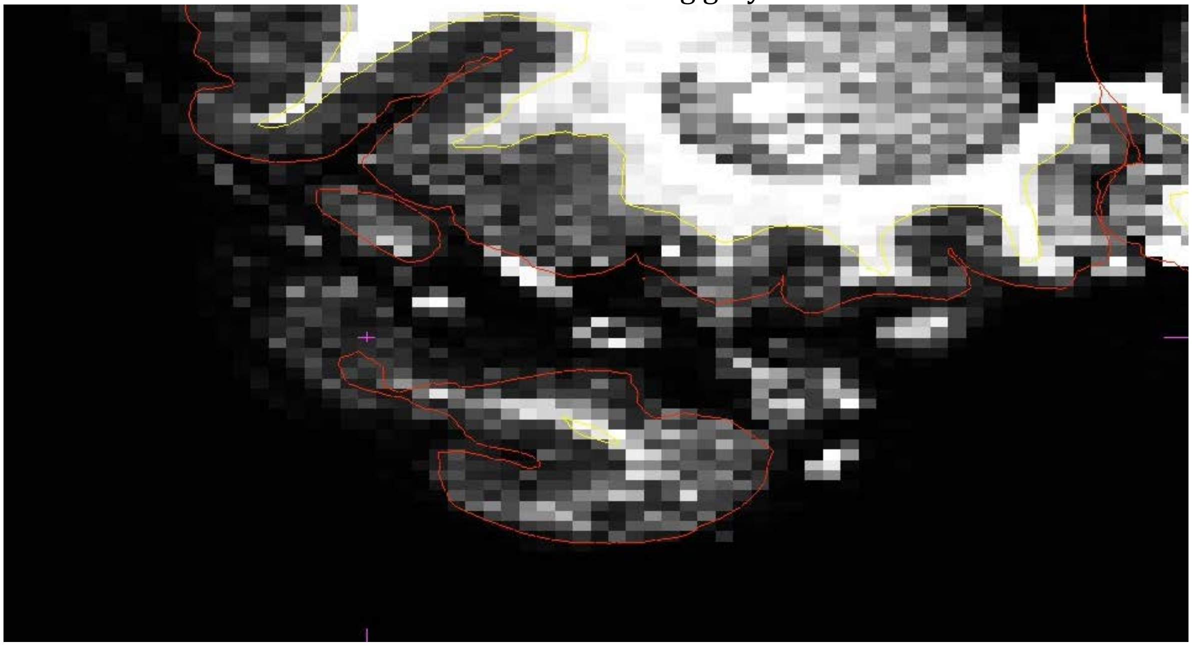

Example 1 – The pial surface (red) that should include the entire temporal lobe is excluding gray matter in the latter portion of the temporal lobe. This can be fixed by finding prominent white matter tracts on nearby slices (in this case anterior slices) and placing control points to manipulate the pial surface out, as seen below.

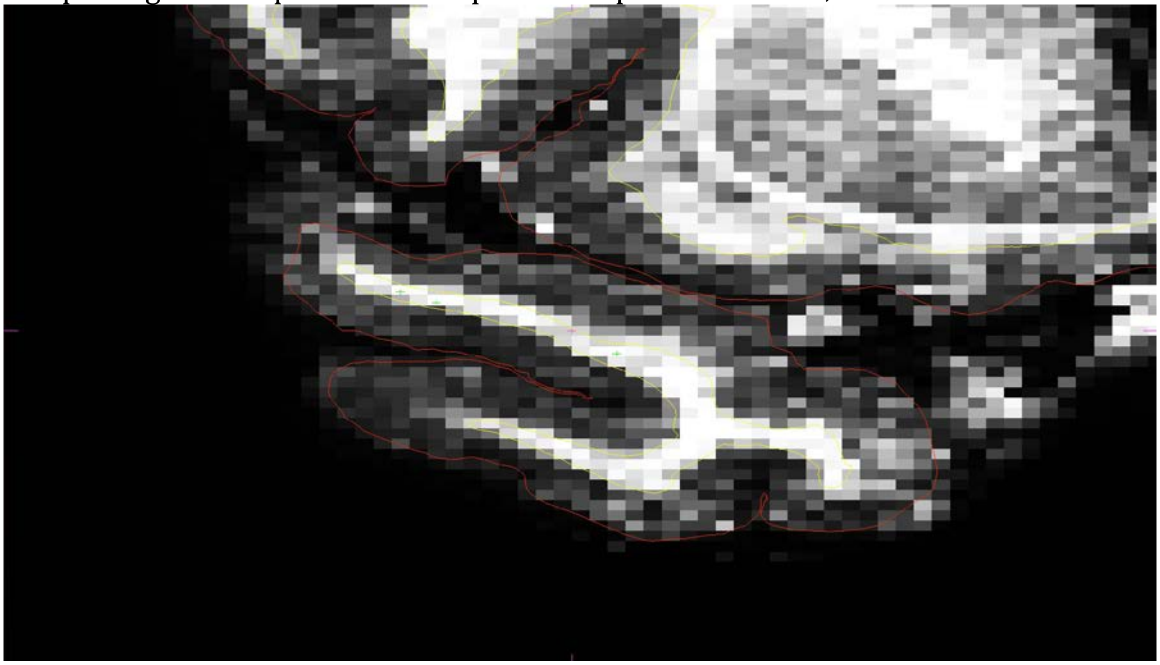

Pial Surface Includes Non-Gray Matter

When the Pial (red) surface includes Non-Gray Matter, it is necessary to erase the erroneously included voxels in the brain mask.

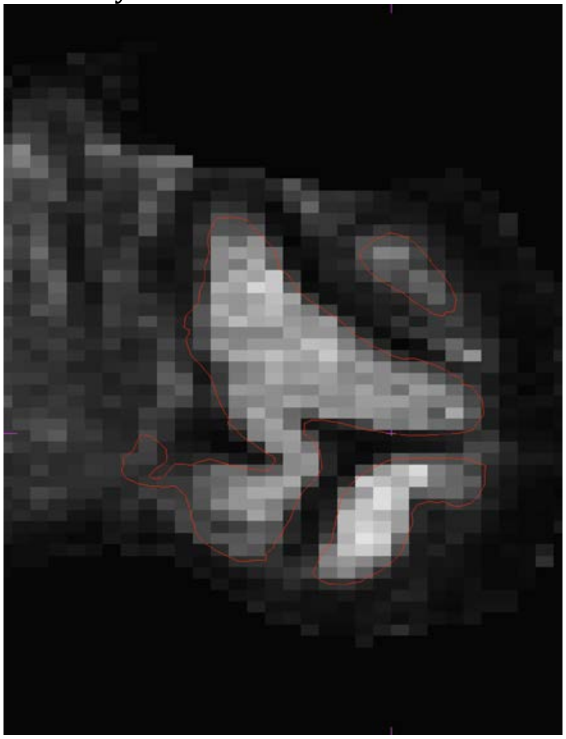

Example 1 – In the medial inferior section of the occipital lobe, the pial surface includes sagittal sinus, which should be excluded. To fix this problem, erase the voxels that should be excluded on the brainmask.mgz, it is not necessary to put down control points.

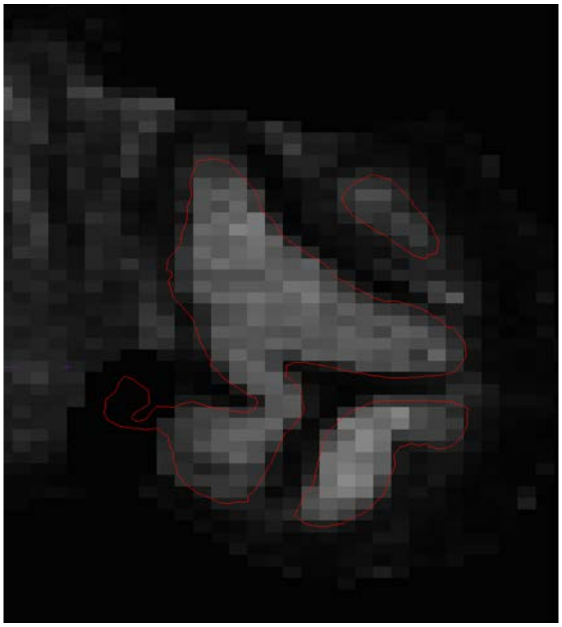

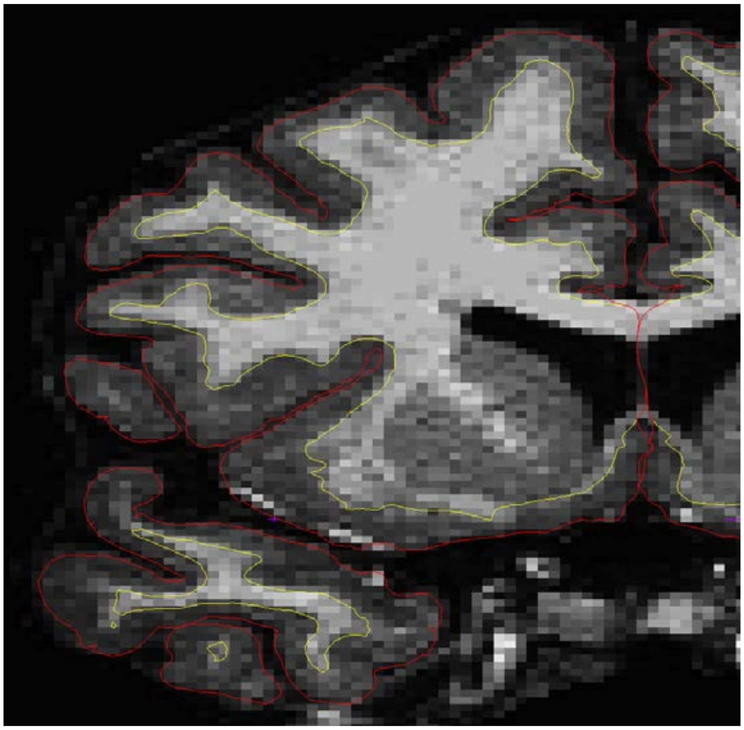

Example 2 – The above example shows the pial surface including non-brain matter near the (readers) left anterior temporal lobe. The non-brain matter is [structure name?] noticeable because of it's distinct higher intensity gradient than the gray matter adjacent to it. This can be erased by changing your target to 'main' in the brush info box, and removing the pixels from the brain mask (below).

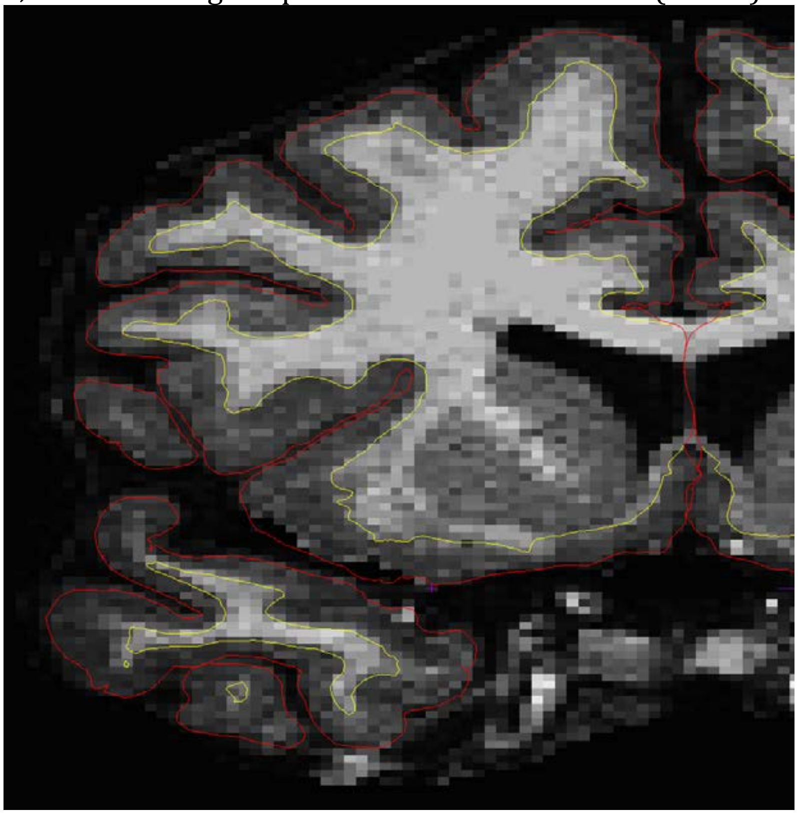

Bridge

Occasionally the white matter surface will connect across gray matter, this type of error is aptly referred to as a bridge. To fix a bridge error, erase the white matter underlying the problem, and put control points on the white matter tracts that are adjacent to the bridge.

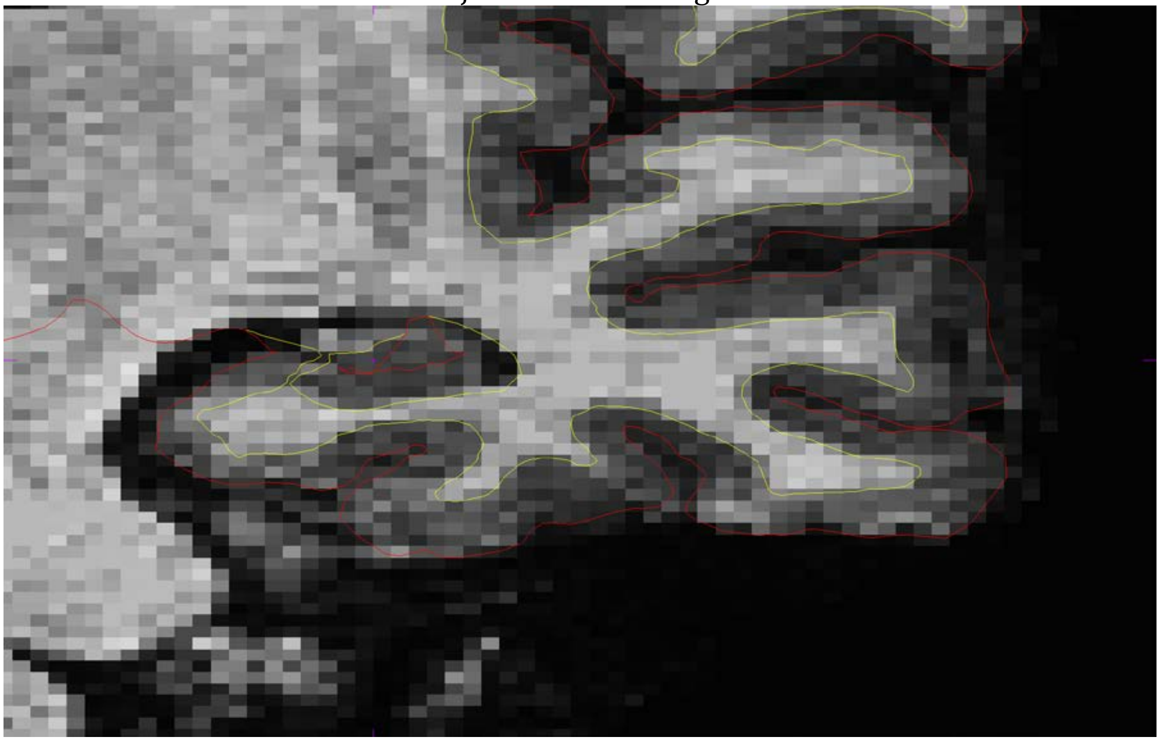

Example 1 – In the above example, a bridge is present between the parahippocampal gyrus and the white matter tract superior to it. To correct these errors erase the white matter underlying the error and put control points on the parahippocampal gyrus and superior white matter, like the image below.

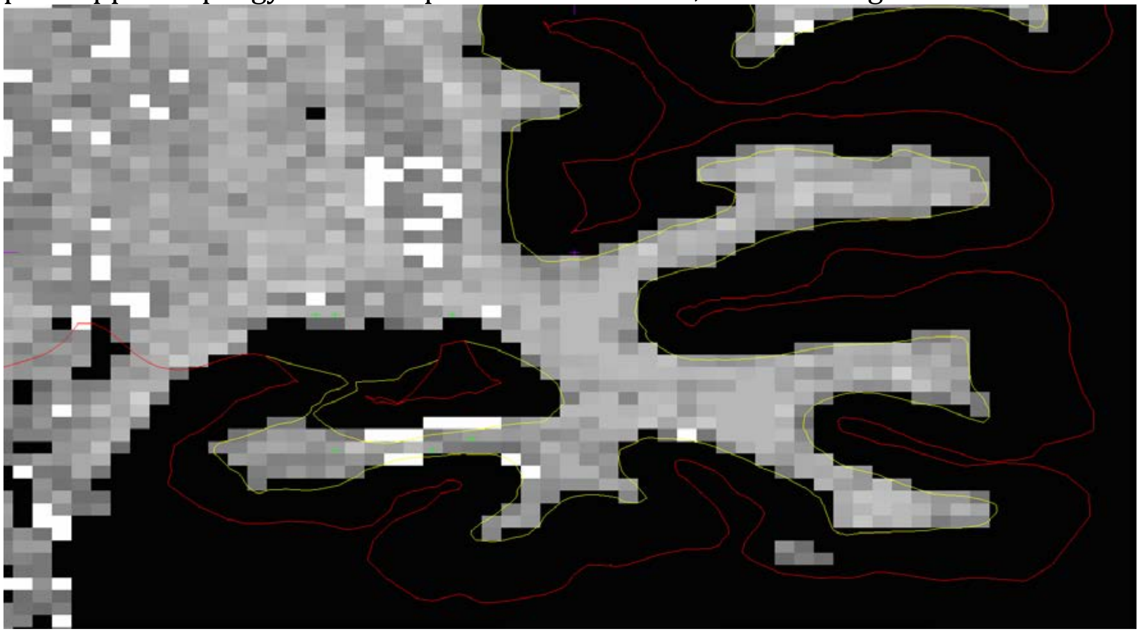

Motion

If a subject moves excessively during the acquisition of the MRI scan, you will see white matter surface artifacts in FreeSurfer. These artifacts are typically present laterally in the brain and can be separate blobs from the white matter surface, or bring the entire surface out.

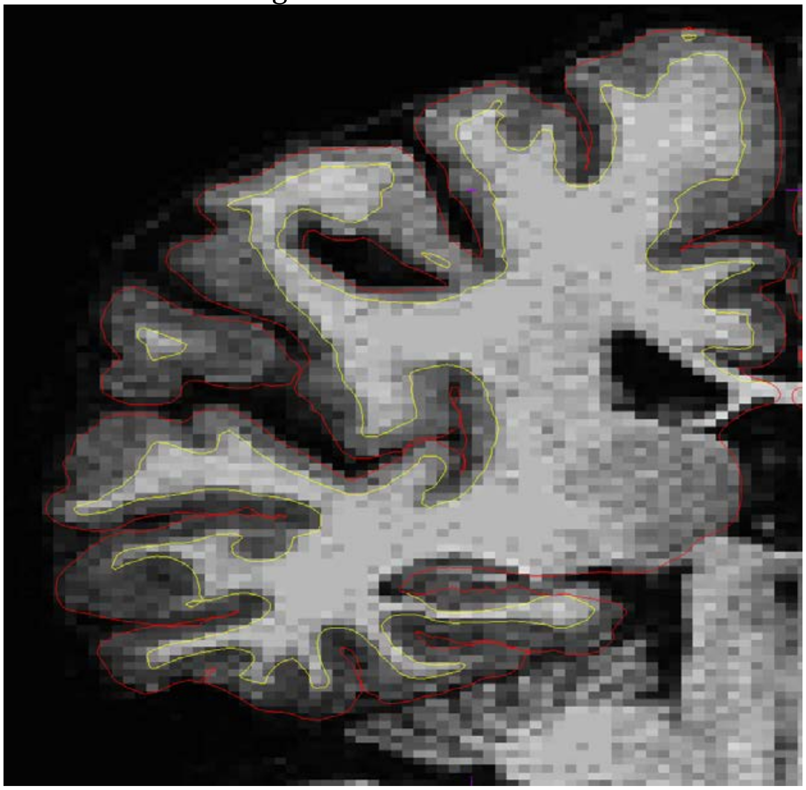

Example 1 – In the above example, you can see motion artifacts in the superior portion of the brain. The first artifact located medially in the brain is separate from the white matter tract. The second artifact is connected to the white matter tract. Both errors should be fixed by erasing the underlying pixels in the white matter mask, and putting down control points (see below).

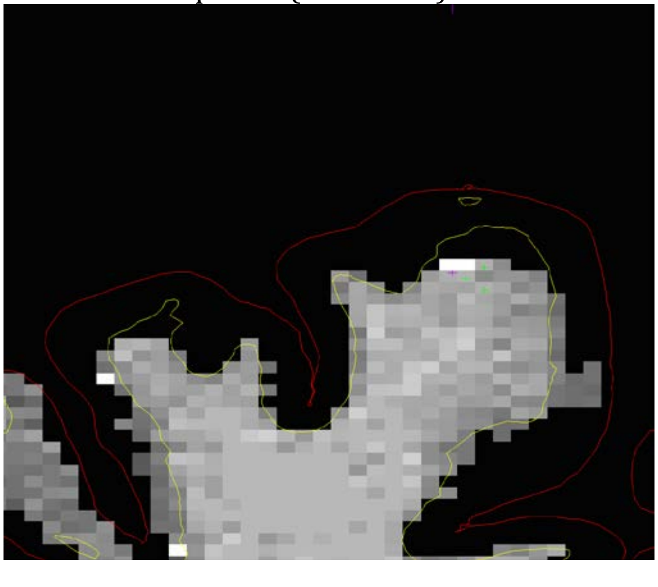

Frontal and Temporal Lobe Specific Editing

The majority of errors are found in the temporal and frontal lobes. When dealing with a subject who has diffuse temporal and frontal lobe errors follow the procedure outlined below, starting with identifying errors.

Identifying Errors

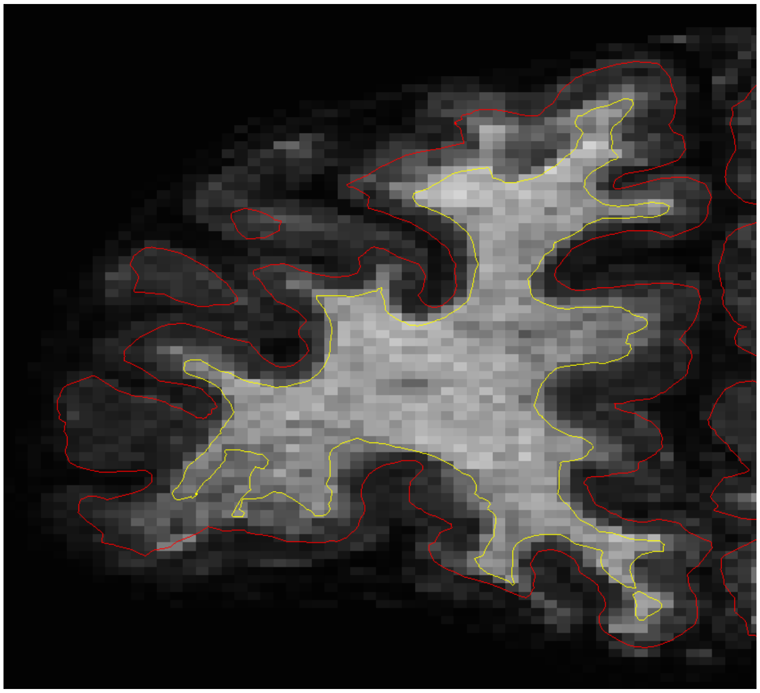

Diffuse Frontal Lobe Errors – In the above picture, the frontal lobe has a myriad of errors that need to be corrected. In the lateral regions, gray and white matter is excluded, and in the medial inferior region, the main surface also excludes white matter.

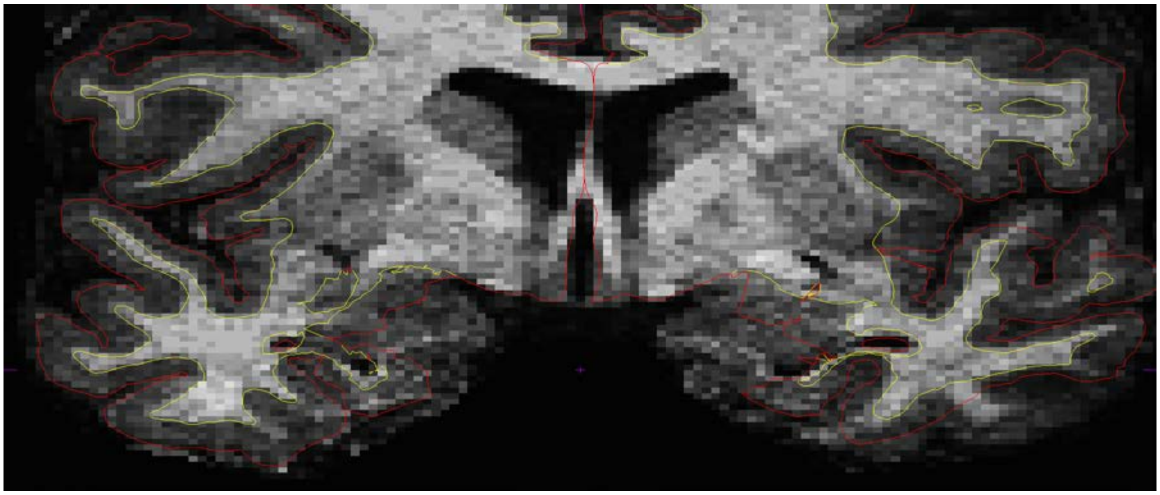

Diffuse Temporal Lobe Errors – Looking at both temporal lobes in the above example, many different errors are preset. Errors include excluded gray matter, excluded white matter, and surface errors.

<u>Skull Stripping</u>

Instead of addressing each error individually, first go through and remove skull around the frontal and temporal lobe, starting towards the end of the temporal lobe. Focus on removing high intensity voxels, and skull that are directly errors. **If unsure about whether a voxel is skull or brain, <u>do not remove.</u>**

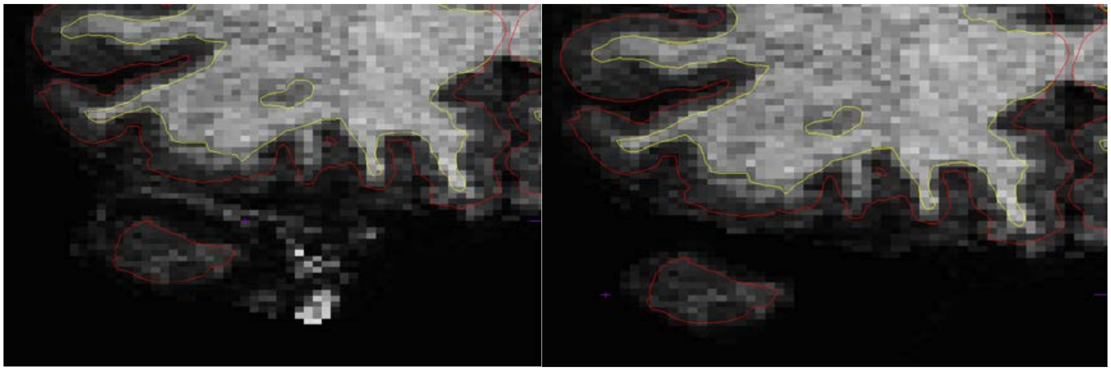

Temporal Lobe Skull Strip – Start skull stripping in the anterior sections of the temporal lobe and concentrate on removing high intensity voxels.

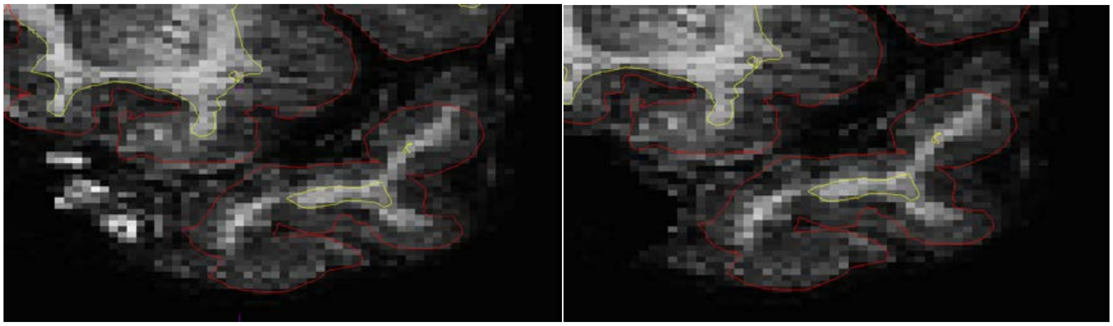

Temporal Lobe Skull Strip – When the border between brain and non-brain matter, err on the side of including too much. Removing high intensity voxels is often enough to improve the overall quality of the temporal and frontal lobes.

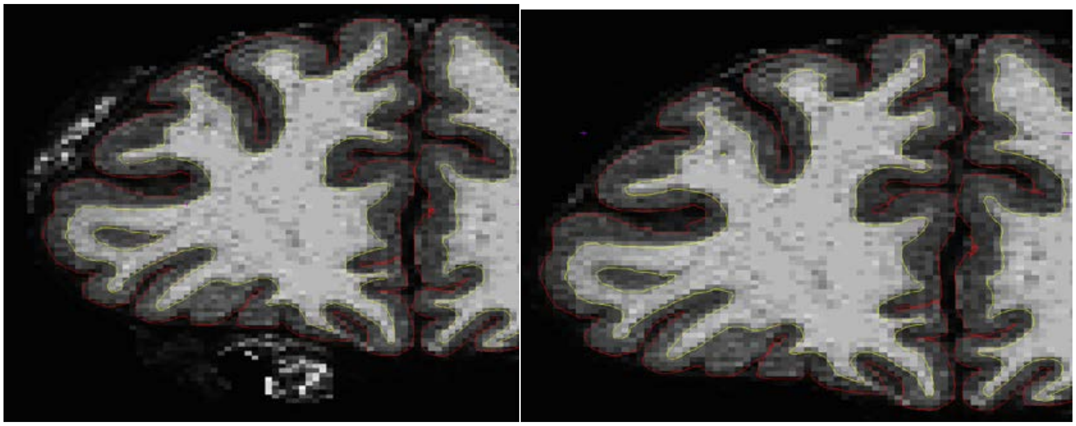

Repeat for every slice, whether there are errors present or not until there is no brain matter present.
